# Using the Pearson’s correlation coefficient as the sole metric to measure the accuracy of quantitative trait prediction: is it sufficient?

**DOI:** 10.3389/fpls.2024.1480463

**Published:** 2024-12-10

**Authors:** Shouhui Pan, Zhongqiang Liu, Yanyun Han, Dongfeng Zhang, Xiangyu Zhao, Jinlong Li, Kaiyi Wang

**Affiliations:** ^1^ Information Technology Research Center, Beijing Academy of Agriculture and Forestry Sciences, Beijing, China; ^2^ National Engineering Research Center for Information Technology in Agriculture, Beijing, China

**Keywords:** genomic selection, quantitative trait prediction, Pearson’s correlation coefficient, evaluation metric, regression prediction

## Abstract

How to evaluate the accuracy of quantitative trait prediction is crucial to choose the best model among several possible choices in plant breeding. Pearson’s correlation coefficient (PCC), serving as a metric for quantifying the strength of the linear association between two variables, is widely used to evaluate the accuracy of the quantitative trait prediction models, and generally performs well in most circumstances. However, PCC may not always offer a comprehensive view of predictive accuracy, especially in cases involving nonlinear relationships or complex dependencies in machine learning-based methods. It has been found that many papers on quantitative trait prediction solely use PCC as a single metric to evaluate the accuracy of their models, which is insufficient and limited from a formal perspective. This study addresses this crucial issue by presenting a typical example and conducting a comparative analysis of PCC and nine other evaluation metrics using four traditional methods and four machine learning-based methods, thereby contributing to the improvement of practical applicability and reliability of plant quantitative trait prediction models. It is recommended to employ PCC in conjunction with other evaluation metrics in a targeted manner based on specific application scenarios to reduce the likelihood of drawing misleading conclusions.

## 1 Introduction

Quantitative trait prediction is receiving increasing attention in plant breeding in recent years (Jeong et al., 2020). It aims to obtain accurate predictions of unobserved genetic or phenotypic values through the integrated analysis of multi-source data (e.g., genomics, phenomics, and enviromics) (Xu et al., 2022). In recent years, machine learning techniques have been introduced and applied in genomic prediction due to their ability to capture various complex potential interactions, non-linear and non-additive effects (Yan et al., 2021; Xu et al., 2022; Li et al., 2024). Specifically, many machine learning-based methods represented by deep neural networks, have been introduced as superior alternatives to traditional linear models (Wang et al., 2023). Evaluating the prediction accuracy is crucial for choosing the best model among several possible choices (Blondel et al., 2015). Pearson’s correlation coefficient (PCC), serving as a metric for quantifying the strength of the linear association between two variables, is widely used to evaluate the accuracy of the quantitative trait prediction models, and generally performs well in most circumstances (Blondel et al., 2015). However, it should be noted that PCC may not always provide a complete picture of predictive accuracy and is flawed for the purpose of method comparison (McGrath et al., 2024), especially in cases involving nonlinear relationships or complex dependencies (González-Recio et al., 2014), the use of inappropriate models, and insufficient model training. It has been found that many papers on quantitative trait prediction based on machine learning solely use PCC as a single metric to evaluate the accuracy of their models, which is insufficient and limited from a formal perspective (González-Recio et al., 2014). Indeed, this issue is not restricted to machine learning-based models for quantitative trait prediction, potentially surfacing in any predictive modeling framework. In certain instances, relying solely on PCC for accuracy evaluation may lead to misleading conclusions (Bland and Altman, 1986; McGrath et al., 2024). Firstly, PCC only measures the overall linear correlation between all observed and predicted values without considering the prediction bias or variance of the model (González-Recio et al., 2014; Abdollahi-Arpanahi et al., 2020). Secondly, PCC measures the strength of a relation between observed and predicted values, not the agreement between them (Bland and Altman, 1986). Thirdly, the PCC value depends on the range or variability of the variables. High variability and a larger sample size tend to provide a more accurate and reliable estimate of the linear relationship between the variables. Conversely, low variability or a narrow range of values can make the correlation coefficient less informative, potentially leading to misleading interpretations. Thus, predictive models selected solely based on PCC metric often fail to align with many practical application scenarios. For example, in practical crop breeding, breeders focus more on the hit rate of head or tail breeding lines rather than the overall correlation in order to select the top-K individuals or eliminate the bottom-K individuals in the ranking. Thus, relying solely on PCC for choosing predictive models makes it difficult to accurately select the top individuals with the highest breeding value (Blondel et al., 2015).

## 2 Limitations of relying solely on PCC for accuracy evaluation

Most often, a common approach to measure the performance of a quantitative model is to plot the scatter diagram of predicted and observed values, and fit them using a simple linear regression model $Y_{observed}=aY_{predicted}+b$ and then compare slope and intercept parameters with the 1:1 line (Piñeiro et al., 2008). In this simple linear regression, if the least squares method is used for parameter estimation, the square of the PCC value between the independent variable and dependent variable (corresponding to the predicted values and observed values in the original quantitative prediction model, respectively) is exactly equal to the R^2^ score of this simple linear regression model (not the R^2^ score of the original quantitative prediction model). This may be one of the reasons why PCC between predicted and observed values is often used in many papers to measure the performance of a quantitative prediction model. There seem to be no issues whatsoever, but the reality is somewhat different. The correlation between predicted and observed values depends on their variability (e.g. range) and distribution (Bland and Altman, 2003). In particular, a change in the scale of the predicted value (e.g. all being multiplied by a certain factor) does not alter the PCC value, but it undoubtedly impacts the performance of a model (Bland and Altman, 1986). For example, if the predicted values are consistently tenfold the observed values, employing the aforementioned simple linear regression model would yield an impeccable straight line characterized by a slope of 10.0 and a PCC value of 1.0. If the ranges of observed and predicted values differ or if there is a non-linear relationship between them due to various factors, such as inherent defects of the prediction model, insufficient model training, substantial differences in data distribution between the test set and the training set, relying solely on the PCC to measure the accuracy of the prediction model may lead to misleading conclusions. Thus, it is not rigorous to solely use PCC as a single metric to measure the prediction accuracy of a model in some published papers.

**Figure 1:**
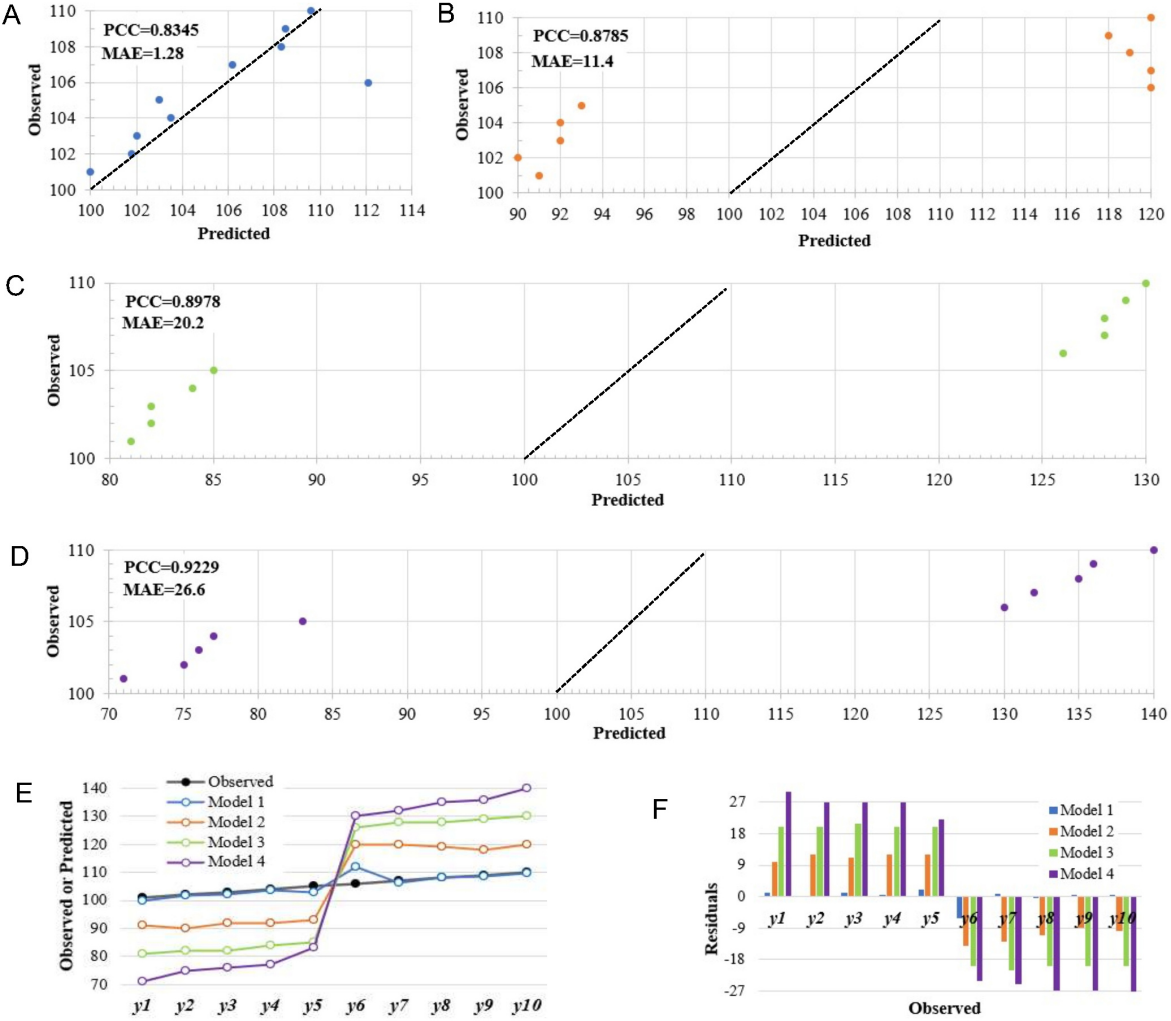
An example of how relying solely on PCC for accuracy evaluation in quantitative trait prediction may lead to misleading conclusions based on the simulated data. **(A-D)** Scatter diagrams of predicted versus observed values for the Model 1, Model 2, Model 3 and Model 4; **(E)** Comparison of observed values with predictions from four models; **(F)** A comparison of residuals among the four models. MAE, mean absolute error; RMSE, root mean squared error; PCC, Pearson’s correlation coefficient.

Here, we present a simple example to elucidate this issue. Suppose our objective is to utilize genotypic and environmental data to forecast the phenotypes of a quantitative trait (e.g., yield). In this scenario, we employ four machine learning-based models individually to make predictions, thereby obtaining the corresponding predicted values for each model. To simplify, let us assume that the test set comprises 10 test data, the details of the input data and four prediction models are omitted here. The observed values and predicted values of each model are shown in **Supplementary Table S1**. The scatter plots of predicted *vs*. observed values and the residual plots are presented in this example to visually assess the prediction accuracy of these four models (**Figure 1**). The PCC between predicted values and observed values in the four models are 0.8345, 0.8785, 0.8978, and 0.9229 respectively. For model 1, except for two data points with residual values of -6.1 and 2, the absolute residuals for the remaining eight data points are all within 1, resulting in a MAE (Mean Absolute Error) of 1.28. In model 2, the absolute values of all residuals are greater than or equal to 10, with a MAE of 11.40. Similarly, in model 3 and model 4, the absolute residuals are relatively larger, with a MAE of 20.20 and 26.60 respectively. Compared with the first three models, the residual value of model 4 fluctuates more widely. If we solely rely on the PCC metric to assess the predictive accuracy of the four models, it would seemingly suggest that Model 1 exhibits the poorest predictive performance while Model 4 displays the most superior accuracy. However, it is patently clear that this conclusion fails to align with the actual situation. Obviously, Model 1 has the smallest residuals among these four models, and shows a better fitting capability to the observed values (**Figures 1E, F**). However, its PCC value (0.8345) is the lowest, which is less than the values of Model 2 (0.8785), Model 3 (0.8978), and Model 4 (0.9229) (**Supplementary Table S1**). This suggests that a model boasting a higher PCC does not always guarantee superior predictive accuracy. This issue may also emerge in the phenotype prediction of real-world crop breeding datasets (**Supplementary Tables S2**-**S39**).

## 3 Empirical analysis based on real-world breeding datasets

**Figure 2:**
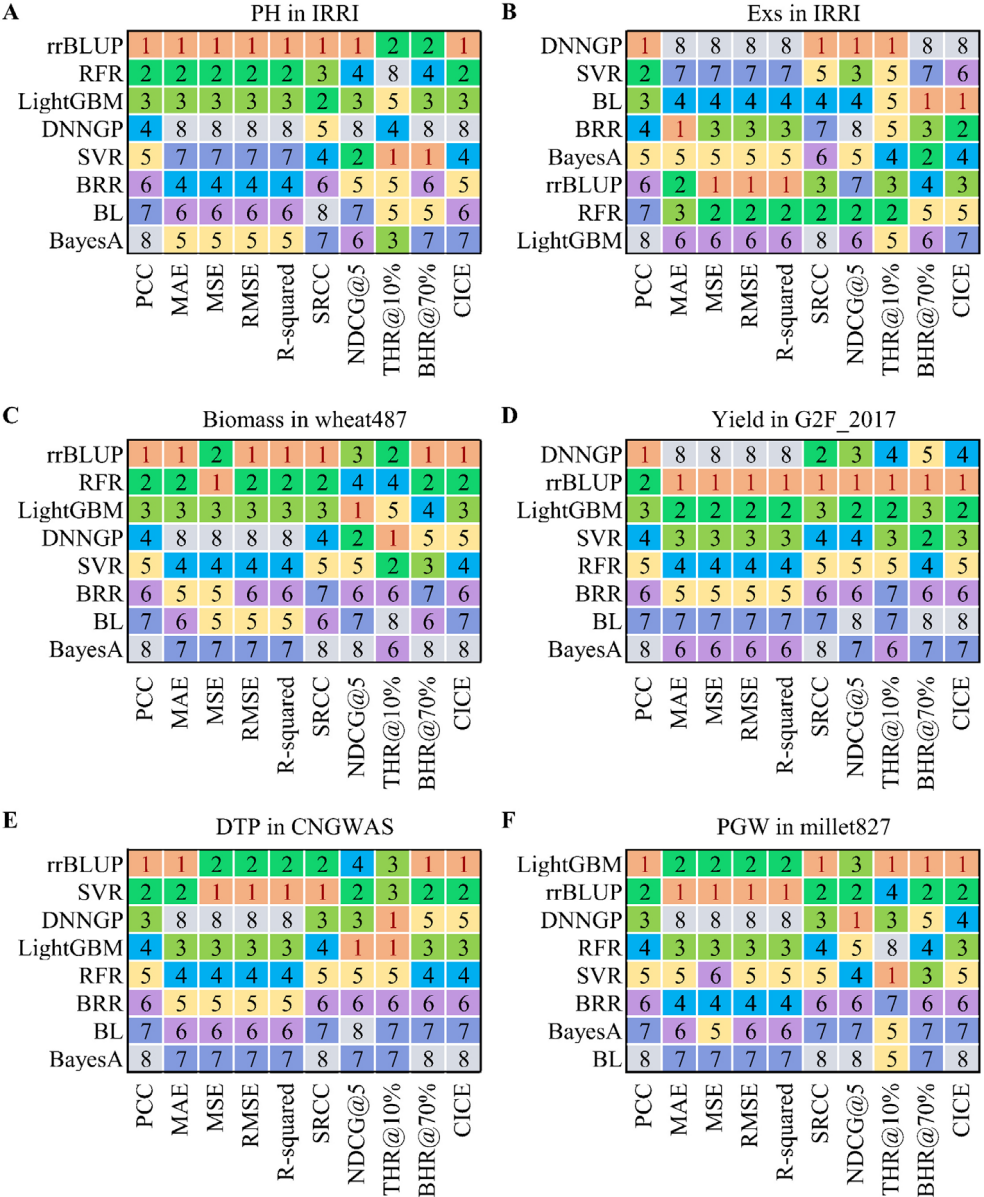
Comparison of the ranking between PCC and nine other evaluation metrics in some phenotype predictions based on real-world breeding data. **(A)** Prediction of the trait PH on the IRRI dataset; **(B)** Prediction of the trait Exs on the IRRI dataset; **(C)** Prediction of the trait Biomass on the wheat487 dataset; **(D)** Prediction of the trait Yield on the G2F_2017 dataset; **(E)** Prediction of the trait DTP on the CNGWAS dataset; **(F)** Prediction of the trait PGW on the millet827 dataset. PH, plant height; Exs, panicle exertion rate; DTP, days to pollen; PGW, per plant grain weight. PCC, Pearson’s correlation coefficient; MAE, mean absolute error; MSE, mean squared error; RMSE, root mean squared error; R-squared, coefficient of determination R²; SRCC, Spearman’s rank correlation coefficient; NDCG@5, top 5 normalized discounted cumulative gain; THR@10%, top 10% hit rate; BHR@70%, bottom 70% hit rate; CICE, combined index for correlation and error; rrBLUP, ridge regression best linear unbiased prediction; BL, Bayesian LASSO; BRR, Bayesian ridge regression; LightGBM, light gradient boosting machine; SVR, support vector regression; RF, random forest; DNNGP, deep neural network for genomic prediction.

To strengthen the persuasiveness and thoroughness of our opinion, the differences between PCC and nine other evaluation metrics were compared by eight methods on seven real-world crop breeding datasets representing different species, traits, sample sizes, and data distributions in this study (**Supplementary Figures S1**-**S14**). These nine metrics were MAE, MSE (Mean Squared Error), RMSE (Root Mean Squared Error), R-squared, SRCC (Spearman’s rank correlation coefficient), NDCG@K (top-K normalized discounted cumulative gain) (Blondel et al., 2015), THR@P% (top-P percent hit ratio), BHR@P% (bottom-P percent hit ratio), and CICE (combined index for correlation and error) (**Supplementary Equation S1**). The eight methods used in this study include: four traditional methods — ridge regression best linear unbiased prediction (rrBLUP), BayesA, Bayesian LASSO (BL), and Bayesian ridge regression (BRR); and four machine learning methods — light gradient boosting machine (LightGBM), support vector regression (SVR), random forest (RF), and deep neural network for genomic prediction (DNNGP) (Wang et al., 2023). Two evaluation schemes, 10-fold cross-validation and one-time test (80% for training and 20% for testing), were involved in this study for comparing the performance of eight models on different datasets. The experimental results indicate that, in some cases, the ranking of the PCC metric of the model is inconsistent with the ranking of other metrics (**Supplementary Tables S2**-**S39**). For example, in the prediction of plant height (PH) using 10-fold cross-validation on the IRRI dataset (Spindel et al., 2015), the PCC score of the DNNGP model (Wang et al., 2023) is higher than those of the BRR and SVR models, with PCC scores of 0.351, 0.347 and 0.211, respectively (**Supplementary Tables S2**). However, the DNNGP model presents a distinctly lowest R² score and ranking compared to the BRR and SVR models, and the ranking of its other metrics, such as MAE, MSE, and RMSE, are also higher than those of the other two models (**Figure 2A**). The potential reason for this phenomenon could lie in the inherent challenges faced by neural network models in fully harnessing their strengths when confronted with relatively small size datasets (Abdollahi-Arpanahi et al., 2020). Similar phenomena also can be observed in the prediction of quantitative traits such as flowing date (FLW), peduncle length (PedL), grain width (GrW), grain yield (YLD), panicle exertion rate (Exs), and lodging score (Lg) on the IRRI dataset (**Supplementary Tables S3**-**S8**, **S22**-**S27**), as well as the average grain yield (GY) on the wheat599 (McLaren et al., 2005) dataset with 1279 markers (**Supplementary Tables S9**, **S28**). Furthermore, in-depth case studies focusing on specific traits of utmost importance in crop breeding, were conducted on five additional datasets including wheat487 (Garcia et al., 2019), G2F_2017 (McFarland et al., 2020), CNGWAS (Yang et al., 2014), USNAM (Li et al., 2024), and millet827 (Wang et al., 2022), further reaffirm and strengthen the aforementioned findings (**Figure 2**; **Supplementary Tables S11**-**S20**, **S30**-**S39**). In these typical cases (**Figures 2C, D**), if we rely solely on PCC metric to measure the accuracy of a model, it will lead to misleading conclusions. On the other hand, the PCC metric exhibits almost consistency with other metrics across all eight models on the wheat599 dataset with 251 principal components after dimensionality reduction (**Supplementary Tables S10**, **S29**). Considering that the phenotypes on the wheat599 dataset is close to a standard normal distribution (**Supplementary Figures S2**, **S9**), and that the features in its genotypes are linearly independent of each other after dimensionality reduction, this implies that the redundant features of genotypes and the data distribution of phenotypes may have a considerable influence on the PCC performance of the model.

A higher PCC value for a prediction model merely indicates a stronger linear correlation between the predicted and observed values, but it does not necessarily imply that the prediction error is smaller. In some scenarios, there may be high PCC values accompanied by high prediction bias (**Supplementary Tables S3**-**S9**, **S11**-**S28**, **S30**-**S40**). Moreover, the PCC value has volatile and opaque characteristics in predictive models based on nondeterministic effects alone, showing noticeable fluctuations across varying test set sizes, distinct random partitions of the data, and even with different random initializations (Ubbens et al., 2021). For example, the PCC value is more susceptible to factors such as sample size and test set size compared to MAE and RMSE (**Supplementary Figure S15**). As the sample size gradually increases, the PCC value shows a more rapidly increasing trend compared to the MAE and RMSE values (**Supplementary Figures S15A–C**). Given a fixed training set, the PCC score exhibits larger fluctuations compared to MAE and RMSE when the size of the test set is small, and tends to decrease and become more stable as the size of the test set gradually increases (**Supplementary Figures S15D–F**). In some cases, such as when the predicted values and observed values are collinear, even if the PCC of the model is high, there may be other issues such as overfitting (Blondel et al., 2015). In practice, in the evaluation of the model, metrics such as MAE, MSE, RMSE, and R² score are more frequently used alternatives to PCC (Scikit-learn, 2023; **Supplementary Figure S16**). Thus, data transformation or standardization is also very important for the objective evaluation of model accuracy. In addition, PCC has an upper limit (equal to the square root of heritability) when heritability is less than one in genomic prediction (Blondel et al., 2015).

## 4 Conclusion

The PCC may not reflect the accuracy of the model if range or variability of observed and predicted values differ or if there is a non-linear relationship between them due to various factors such as outliers, data distribution, test set size and inappropriate models. For the performance evaluation of the model, it is essential to first test whether there is a linear relationship between the observed and predicted values, along with their variability. If their relationship is not a simple linear regression or if their value ranges differ, the model’s predictions may be not good, then the PCC may not be a useful metric. At this time, using PCC solely as a measure of model accuracy should be approached with caution, as each metric has its own advantages and disadvantages in different application scenarios (González-Recio et al., 2014; **Supplementary Table S41**) and there is no one which can be used solely. When selecting metrics for evaluating the accuracy of the model, multiple factors such as practical application scenarios, redundant features of genotype data, distribution of phenotype data, train-test split of dataset, the size of test set, and model complexity should be comprehensively considered. For example, in crop breeding scenarios, the THR@P%, or BHR@P% may be more suitable for measuring the performance of the model compared to PCC, as breeders are more concerned with how to select the top-K individuals or eliminate the bottom-K individuals. It is recommended to employ a combination of multiple metrics such as MAE, RMSE, R² score, NDCG and root mean squared deviation (RMSD) (Piñeiro et al., 2008) rather than just using the PCC as a sole metric to assess the accuracy of a quantitative trait prediction model. In addition, the Bland–Altman method (Bland and Altman, 2003) and visual assessment such as scatter plot of predicted and observed values are also valuable supplement for evaluating the accuracy of the model (Piñeiro et al., 2008). To improve the operability in practical applications, the clear guidance and detailed steps on how to select and apply evaluation metrics in several typical scenarios are provided (**Supplementary Table S42**). Furthermore, in order to facilitate the comparison of performance among models and minimize the likelihood of misleading conclusions, a combination of PCC and MAE, called combined index for correlation and error (CICE) (**Supplementary Equation S1**) was proposed for model evaluation in general scenarios. Empirical results indicate that CICE effectively balances prediction trend and prediction bias in model evaluation compared to using PCC as the sole measure (**Supplementary Tables S2**-**S40**).

## Data Availability

The original contributions presented in the study are included in the article/**Supplementary Material**. Further inquiries can be directed to the corresponding author.
