# Supplementary material for: Using the Pearson’s correlation coefficient as the sole metric to measure the accuracy of quantitative trait prediction: is it sufficient?
Source: Front Plant Sci. 2024 Dec 10;15:1480463. doi: 10.3389/fpls.2024.1480463 (PMC11667204; doi:10.3389/fpls.2024.1480463)
Supplement: Supplementary file 1 [file DataSheet1.docx]

**Supplementary Material**

1. **Supplementary Datasets**

Seven real-world crop breeding datasets, representing different species, traits, sample sizes, and data distribution, were used in this study: wheat599 (McLaren et al., 2005), IRRI (Spindel et al., 2015), wheat487 (Garcia et al., 2019), G2F_2017 (McFarland et al., 2020), CNGWAS (Yang et al., 2014; Li et al., 2024), USNAM (Buckler et al., 2009) and millet827 (Wang et al., 2022).

The first dataset, wheat599, is a collection of 599 historical wheat lines from the International Maize and Wheat Improvement Center (CIMMYT) Global Wheat Program (McLaren et al., 2005). The number of genotypes and markers in this dataset is 599 and 1279, respectively. The principal component analysis (PCA) method was used to extract the top 95% feature variance of SNP markers (Wang et al., 2023). After data dimension reduction, 251 principal components were retained for analysis. The phenotypic values for the 599 wheat lines were the average grain yield (GY) evaluated in environment 1.

The second dataset, IRRI, consists of 278 lines selected from the International Rice Research Institute (IRRI) irrigated rice breeding program (Spindel et al., 2015). The genotype data is from the post-imputation GBS dataset used specifically for GWAS in Spindel et al., 2015. After removing all monomorphic SNPs, SNPs with call rates < 75%, and SNPs with minor allele frequencies (MAF) < 0.05, 71,710 SNPs were retained. The Phenotype data is from the predicted means for the 2012 dry season phenotype data of 342 breeding lines. After removing breeding lines with missing genotype data, 278 breeding lines were retained for analysis. Seven phenotypic traits are used in this study, including plant height (PH), flowering date (FLW), Peduncle length (PedL), Grain width (GrW), grain yield (YLD), panicle exertion rate (Exs), and lodging score (Lg).

The third dataset, wheat487, is a collection of 487 wheat lines that were selected from an extensive repository of 568 spring wheat accessions hailing from diverse origins across 36 different countries (Garcia et al., 2019; Li et al., 2024). The phenotypes were obtained from two field trials that were conducted in Tarlee, South Australia (34°S, 139° E) under rain-fed conditions during the years 2014 and 2015. Genotyping was performed on each line using the Illumina iSelect 90K SNP array. After quality control for missing data, heterozygosity, and minor allele frequency (MAF>0.05), 30,548 polymorphic markers were retained. Subsequently, through the integration of genomic and phenotypic data, 487 breeding lines were retained (Li et al., 2024). Two traits such as biomass and yield are used in this study. The two important traits such as biomass and yield were focused in this study.

The fourth dataset, G2F_2017, contained 356 tested maize hybrids selected from the 2017 field season of the Maize Genomes to Fields (G2F) project (McFarland et al., 2020; Li et al., 2024). SNPs with a MAF > 5% and a per locus missing rate < 10% were retained, resulting in 21,011 remaining SNPs (Li et al., 2024). This study focused on two important traits: plant height (PH) and yield.

The fifth dataset, CNGWAS, consisted of 508 maize inbred lines, selected from a set of 513 inbred lines that represent global maize diversity with tropical, subtropical, and temperate backgrounds (Yang et al., 2014; Li et al., 2024); these were genotyped with 56,110 SNPs using the MaizeSNP50 BeadChip. After quality control for missing data, and minor allele frequency (MAF>0.05), 21,011 remaining SNPs were retained (Li et al., 2024). Two important traits, days to pollen (DTP) and plant height (PH), were focused on in this study.

The sixth dataset, USNAM, comprised a subset of 5,000 maize recombinant inbred lines (RILs) derived from the maize nested association mapping population of 200 RILs from 25 crosses between diverse inbred lines and B73 (Buckler et al., 2009). A comprehensive evaluation of parents and families was conducted, encompassing 19 quantitative traits across up to 11 diverse environments in 2006 and 2007. After quality control for missing data, and minor allele frequency (MAF>0.05), 21,011 remaining SNPs and 4,505 lines were retained (Li et al., 2024). In this study, two important traits, 100 grain weight (GW100) and plant height (PH) were focused on.

The seventh dataset, millet827, consisted of 827 different foxtail millet cultivars collected from China; these were sequenced and genotyped with 161,562 SNPs (Wang et al., 2022). After quality control for missing data, and minor allele frequency (MAF>0.05), a subset of 64,299 SNPs was retained for case study. Two key traits, main stem height (MSH) and per plant grain weight (PGW) were focused on in this study.

1. **Supplementary Equations**

The PCC serves as a metric for evaluating the accuracy of the model's predicted trend, while the MAE quantifies the magnitude of numerical deviation. Thus, we proposed a novel metric called combined index for correlation and error (CICE), which integrates both predicted trend and numerical deviation to comprehensively evaluate the algorithm's overall performance.

The proposed metric CICE is defined as follows:

$CICE=\alpha\times PCC+(1-\alpha)\times scale(MAE)$ (S1)

where PCC is the Pearson’s correlation coefficient between observed and predicted values, $\alpha$ is the weight of PCC and is set to 0.5 in this paper, $scale(MAE)$ is the normalized version of MAE and is defined as

$scale\left( MAE \right)=\exp\left( -\frac{MAE}{\max\left( Y \right)-\min\left( Y \right)+\epsilon} \right)$ (S2)

where $exp(▪)$ is an exponential function with a base transcendental number e, $\epsilon$ is very small constant (e.g. 1.0e-5) to prevent the divisor from being 0, $\max\left( Y \right)$ is the maximum observed value in the test set, $\min\left( Y \right)$ is the minimum observed value in the test set.

1. **Supplementary Figures**


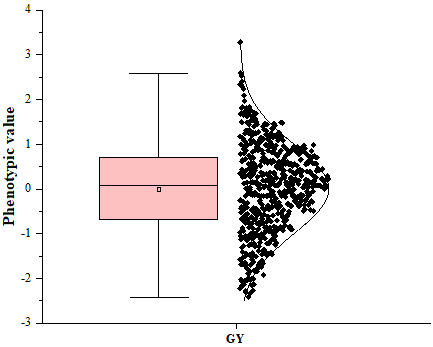


**Supplementary Figure S1.**  Distribution of phenotypic observed values for the trait GY on the wheat599 datasets. GY, average grain yield.


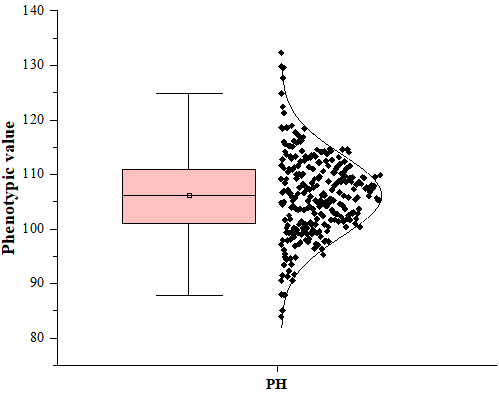

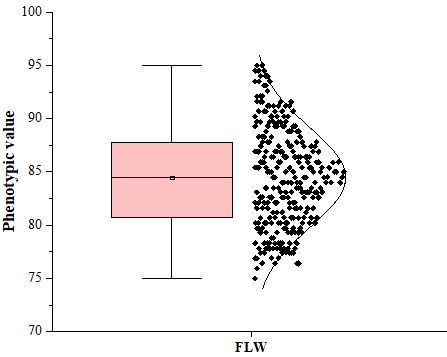


**(A) (B)**


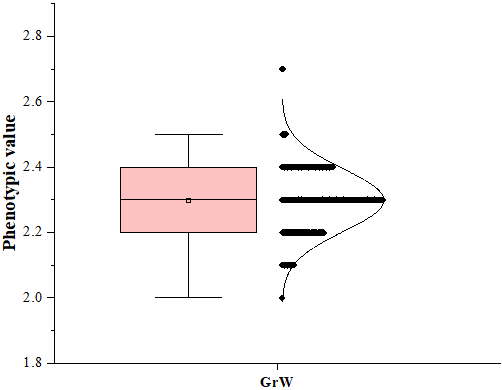

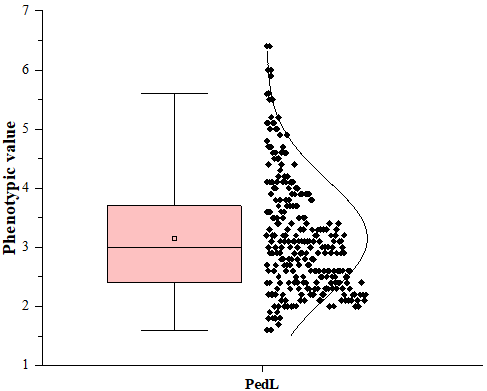


(C) (D)


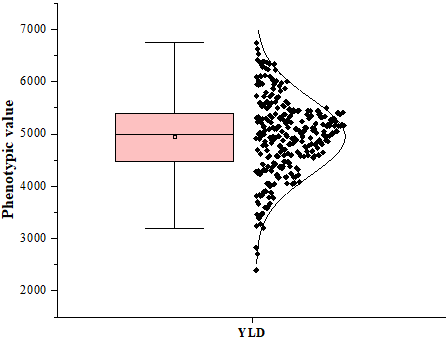

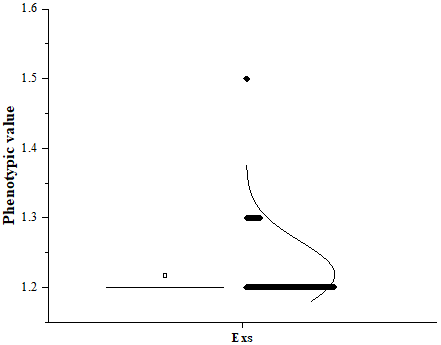


**(E) (F)**


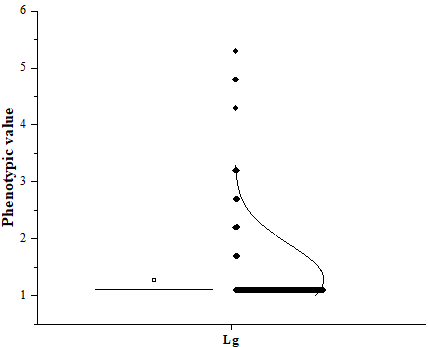


**(G)**

**Supplementary Figure S2.** Distribution of phenotypic observed values for each trait on the IRRI datasets. PH, plant height; FLW, flowering date; PedL, peduncle length; GrW, grain width; YLD, grain yield; Exs, panicle exertion rate; Lg, lodging score.


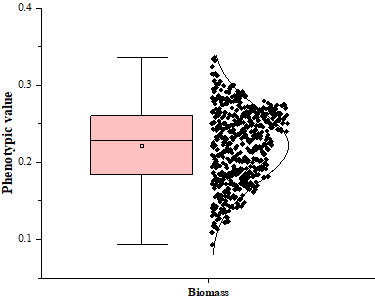

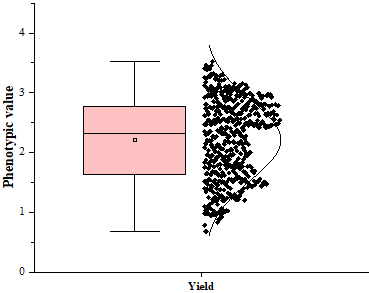


**(A) (B)**

**Supplementary Figure S3.** Distribution of phenotypic observed values for the traits Biomass and Yield on the wheat487 dataset.


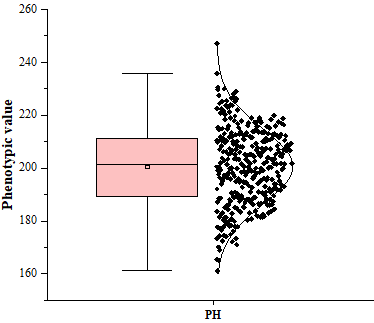

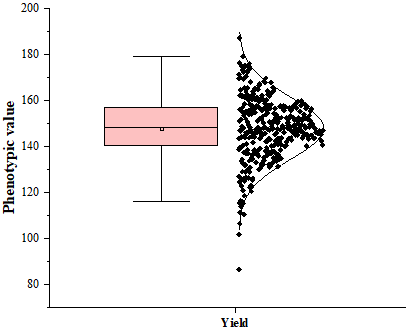


**(A) (B)**

**Supplementary Figure S4.** Distribution of phenotypic observed values of the traits PH and Yield on the G2F_2017 dataset.

PH, plant height.


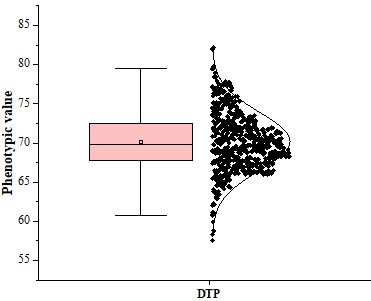

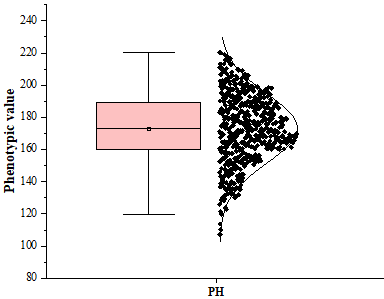


**(A) (B)**

**Supplementary Figure S5.** Distribution of phenotypic observed values for the traits DTP and PH on the CNGWAS dataset.

DTP, days to pollen; PH, plant height.


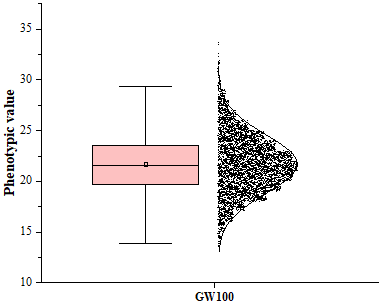

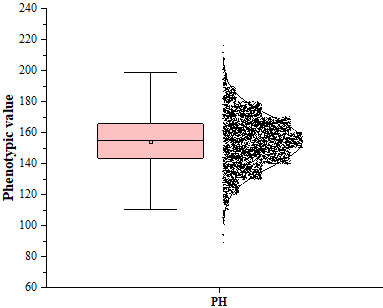


**(A) (B)**

**Supplementary Figure S6.** Distribution of phenotypic observed values for the traits GW100 and PH on the USNAM dataset.

GW100, 100 grain weight; PH, plant height.


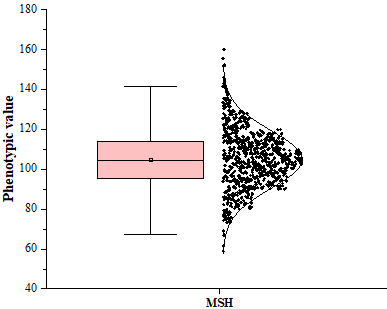

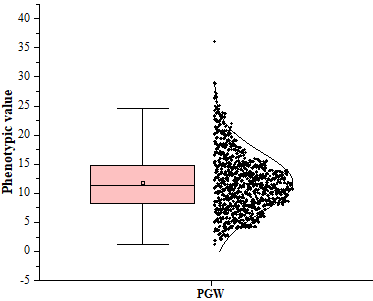


**(A) (B)**

**Supplementary Figure S7.** Distribution of phenotypic observed values for the traits MSH and PGW on the millet827 dataset.

MSH, main stem height; PGW, per plant grain weight.


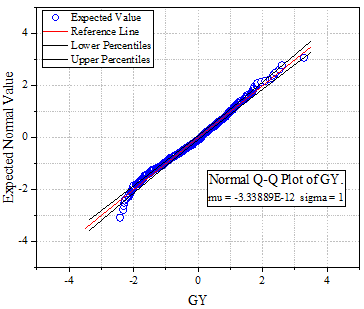


**Supplementary Figure S8.** Normal Q-Q plot of observed values for the traits GY on the wheat599 datasets. GY, average grain yield.


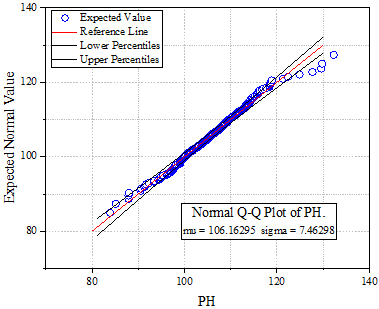

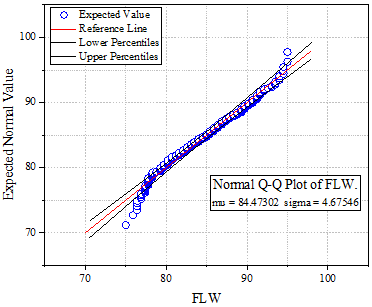


**(A) (B)**
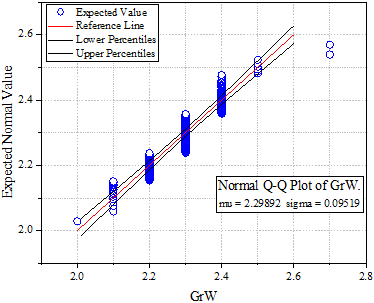

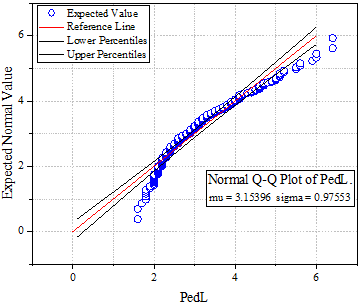


**(C) (D)**
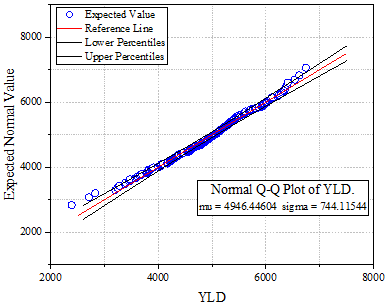

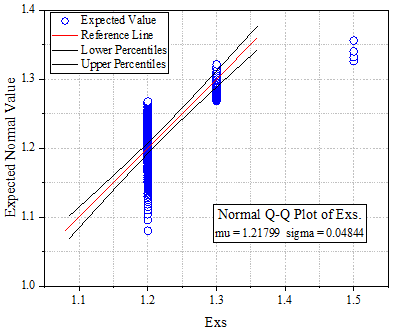


**(E) (F)**


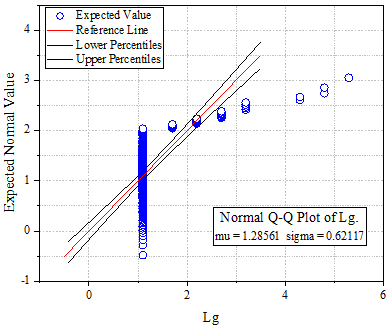


**(G)**

**Supplementary Figure S9.** Normal Q-Q plot of phenotypic observed values for each trait on the IRRI dataset.

FLW, flowering date; PedL, peduncle length; GrW, grain width; YLD, grain yield; Exs, panicle exertion rate; Lg, lodging score.


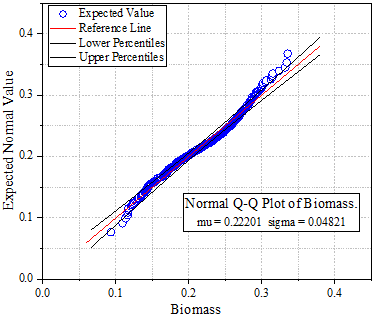

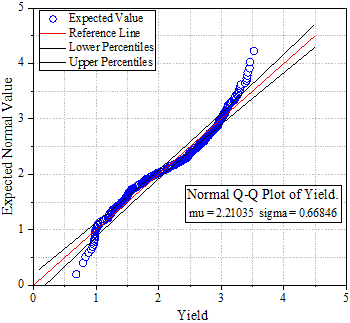


**(A) (B)**

**Supplementary Figure S10.** Normal Q-Q plot of phenotypic observed values for the traits Biomass and Yield on the wheat487 dataset.


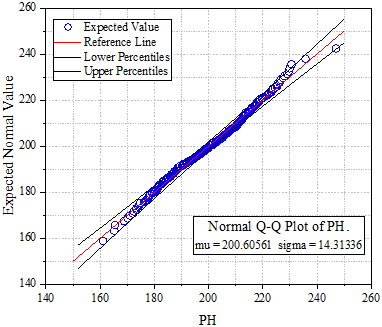

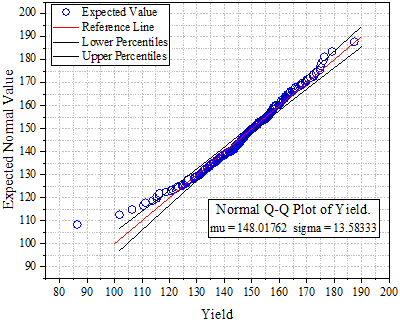


**(A) (B)**

**Supplementary Figure S11.** Normal Q-Q plot of phenotypic observed values for the traits PH and Yield on the G2F_2017 dataset.

PH, plant height.


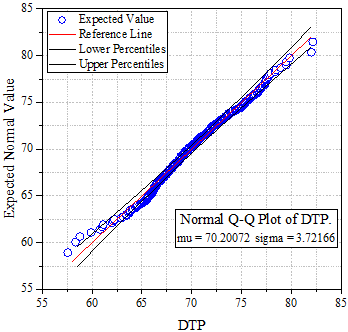

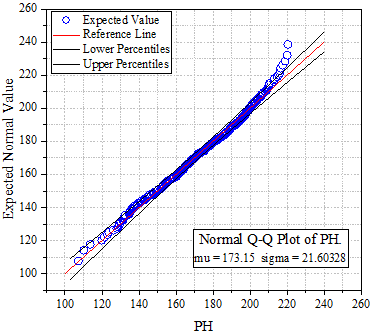


**(A) (B)**

**Supplementary Figure S12.** Normal Q-Q plot of phenotypic observed values for the traits DTP and PH on the CNGWAS dataset.

DTP, days to pollen; PH, plant height.


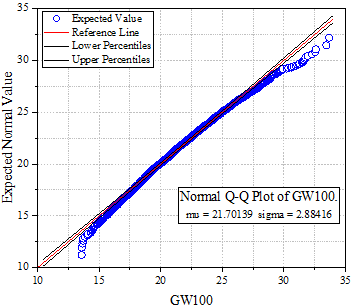

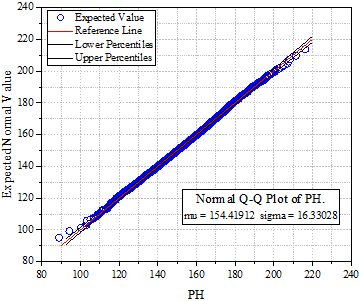


**(A) (B)**

**Supplementary Figure S13.** Normal Q-Q plot of phenotypic observed values for the traits GW100 and PH on the USNAM dataset.

GW100, 100 grain weight; PH, plant height.


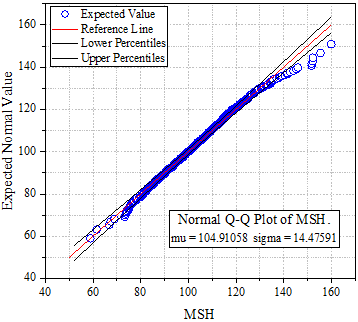

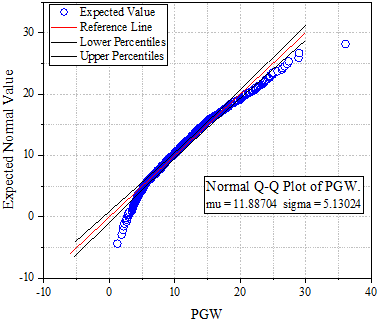


**(A) (B)**

**Supplementary Figure S14.** Normal Q-Q plot of phenotypic observed values for the traits MSH and PGW on the millet827 dataset.

MSH, main stem height; PGW, per plant grain weight.


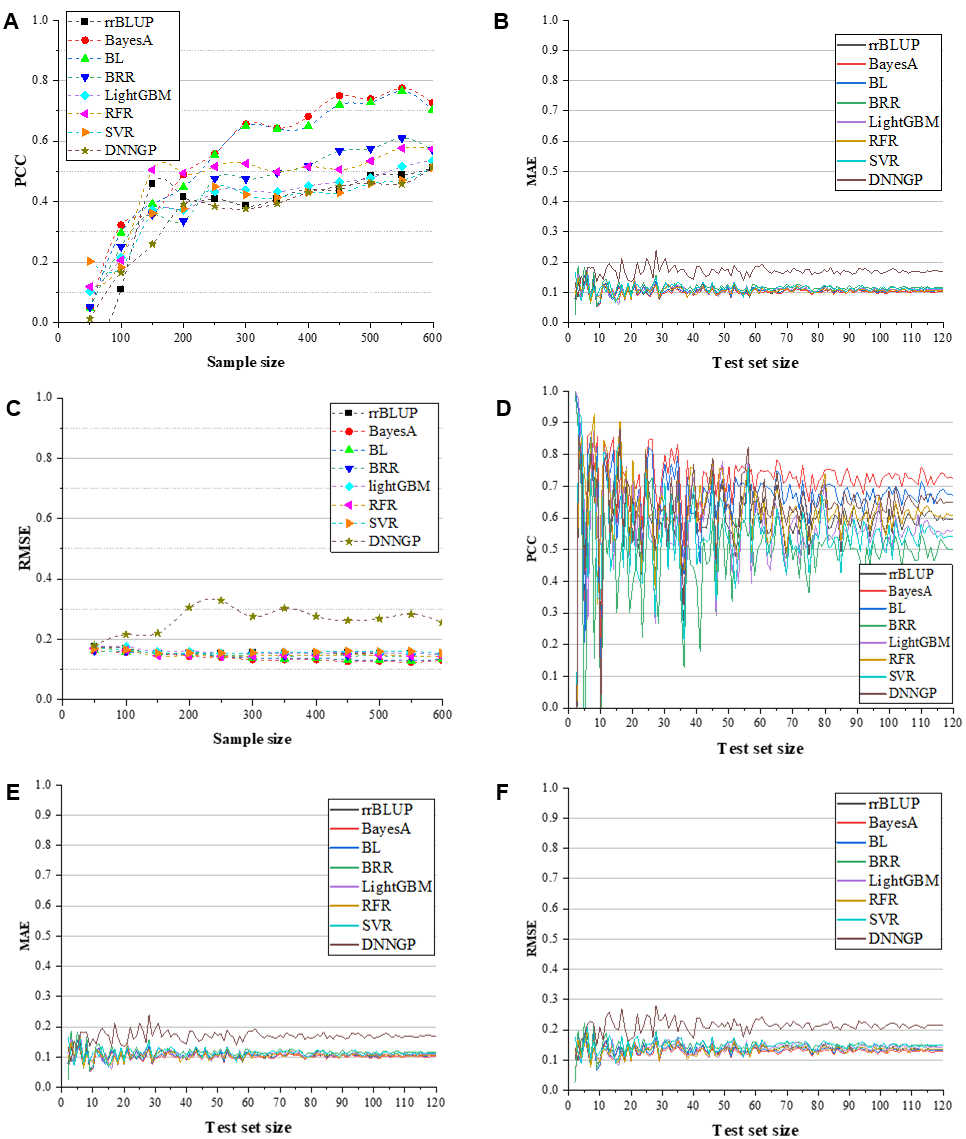


**Supplementary Figure S15.** The effect of sample size and test set size on the values of PCC, MAE, and RMSE on the wheat599 dataset. **(A–C)** The effect of sample size on the values of PCC **(A)**, MAE **(B)**, and RMSE **(C)** through 10-fold cross validation on the wheat599 dataset; **(D–F)** the effect of test set size on the values of PCC **(D)**, MAE **(E)**, and RMSE **(F)** with a fixed training set size on the wheat599 dataset. The sample size of the training set (479 lines) accounts for 80% of the dataset, and the test samples were randomly selected from the remaining 120 lines. For the convenience of comparison between metrics, the MAE in Figures **(B)** and **(E)**, and RMSE in Figures **(C)** and **(F)** were normalized, i.e. $t^{'}=t/(\max\left( Y \right)-\min\left( Y \right))$, where $t^{'}$ is the normalized value of the metric, $t$ is the original value of the metric, $max(Y)$ is the maximum observed value of the predicted phenotype on the dataset, and $min(Y)$ is the minimum observed value of the predicted phenotype on the dataset. PCC, Pearson’s correlation coefficient; MAE, mean absolute error; RMSE, root mean squared error; rrBLUP, ridge regression best linear unbiased prediction; BL, Bayesian LASSO; BRR, Bayesian ridge regression; LightGBM, light gradient boosting machine; SVR, support vector regression; RF, random forest; DNNGP, deep neural network for genomic prediction.


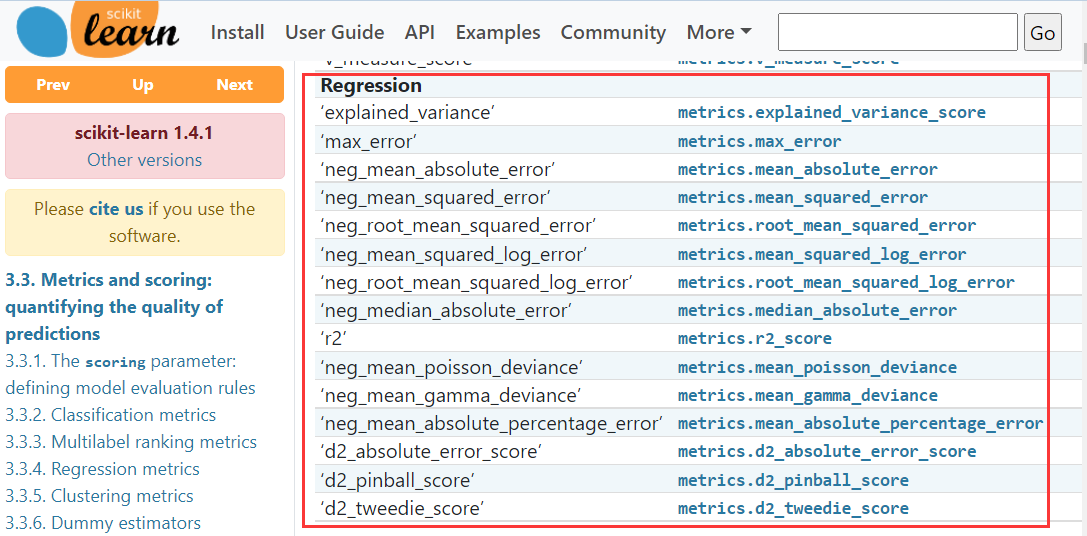


**Supplementary Figure S16.** Regression metrics in machine learning library Scikit-learn.

Scikit-learn is one of the most well-known and widely used machine learning libraries that provides numerous regression algorithms, such as linear regression (LR), decision trees (DT), random forests (RF), and support vector machines (SVM). The Scikit-learn library provides more than 10 regression metrics such as mean absolute error (MAE), mean squared error (MSE), and *R*^2^ score, but not including Pearson’s correlation coefficient. This figure is from Chapter 3.3 of the user guide for Scikit-learn version 1.3.1, with the chapter title “Metrics and scoring: quantifying the quality of predictions” (Scikit-learn, 2023).

Source URL: <https://scikit-learn.org/stable/modules/model_evaluation.html> (Accessed on 15 Dec 2023).

1. **Supplementary Tables**

**Supplementary Table 1.** An example of how relying solely on PCC for accuracy evaluation in quantitative trait prediction may lead to misleading conclusions based on simulated data.

|  |  | ***y*_1_** | ***y*_2_** | ***y*_3_** | ***y*_4_** | ***y*_5_** | ***y*_6_** | ***y*_7_** | ***y*_8_** | ***y*_9_** | ***y*_10_** |  | **Mean** | **MAE** | **RMSE** | **PCC** |
| --- | --- | --- | --- | --- | --- | --- | --- | --- | --- | --- | --- | --- | --- | --- | --- | --- |
| Observed | | 101 | 102 | 103 | 104 | 105 | 106 | 107 | 108 | 109 | 110 |  | 105.50 |  |  |  |
| Model 1 | Predicted | 100 | 101.8 | 102 | 103.5 | 103 | 112.1 | 106.2 | 108.3 | 108.5 | 109.6 |  | 105.50 | 1.28 | 2.11 | 0.8345 |
|  | Residual | 1 | 0.2 | 1 | 0.5 | 2 | -6.1 | 0.8 | -0.3 | 0.5 | 0.4 |  |  |  |  |  |
| Model 2 | Predicted | 91 | 90 | 92 | 92 | 93 | 120 | 120 | 119 | 118 | 120 |  | 105.50 | 11.40 | 11.49 | 0.8785 |
|  | Residual | 10 | 12 | 11 | 12 | 12 | -14 | -13 | -11 | -9 | -10 |  |  |  |  |  |
| Model 3 | Predicted | 81 | 82 | 82 | 84 | 85 | 126 | 128 | 128 | 129 | 130 |  | 105.50 | 20.20 | 20.20 | 0.8978 |
|  | Residual | 20 | 20 | 21 | 20 | 20 | -20 | -21 | -20 | -20 | -20 |  |  |  |  |  |
| Model 4 | Predicted | 71 | 75 | 76 | 77 | 83 | 130 | 132 | 135 | 136 | 140 |  | 105.50 | 26.60 | 26.70 | 0.9229 |
|  | Residual | 30 | 27 | 27 | 27 | 22 | -24 | -25 | -27 | -27 | -30 |  |  |  |  |  |

MAE, mean absolute error; RMSE, root mean squared error; PCC, Pearson’s correlation coefficient.

**Supplementary Table S2.** Score and ranking of eight prediction methods on PCC and nine other evaluation metrics for the trait PH over 10-fold cross validation on the IRRI dataset.

|  | **Method** | **PCC** | **MAE** | **MSE** | **RMSE** | **R-squared** | **SRCC** | **NDCG@5** | **THR@10%** | **BHR@70%** | **CICE** |
| --- | --- | --- | --- | --- | --- | --- | --- | --- | --- | --- | --- |
| Score | rrBLUP | 0.496 | 4.757 | 42.717 | 6.424 | 0.237 | 0.515 | 0.958 | 0.267 | 0.805 | 0.701 |
|  | RFR | 0.458 | 5.025 | 45.219 | 6.621 | 0.188 | 0.455 | 0.941 | 0.200 | 0.789 | 0.680 |
|  | LightGBM | 0.439 | 5.133 | 46.464 | 6.704 | 0.167 | 0.464 | 0.953 | 0.200 | 0.789 | 0.669 |
|  | DNNGP | 0.351 | 26.028 | 1000.259 | 29.845 | -18.862 | 0.361 | 0.931 | 0.233 | 0.700 | 0.468 |
|  | SVR | 0.347 | 6.242 | 141.297 | 10.166 | -1.418 | 0.442 | 0.955 | 0.367 | 0.821 | 0.613 |
|  | BRR | 0.211 | 5.650 | 54.627 | 7.332 | 0.004 | 0.227 | 0.940 | 0.200 | 0.747 | 0.551 |
|  | BL | 0.170 | 6.023 | 62.702 | 7.868 | -0.158 | 0.182 | 0.936 | 0.200 | 0.753 | 0.526 |
|  | BayesA | 0.166 | 5.924 | 60.518 | 7.725 | -0.114 | 0.187 | 0.937 | 0.233 | 0.737 | 0.525 |
| Ranking | rrBLUP | 1 | 1 | 1 | 1 | 1 | 1 | 1 | 2 | 2 | 1 |
|  | RFR | 2 | 2 | 2 | 2 | 2 | 3 | 4 | 8 | 4 | 2 |
|  | LightGBM | 3 | 3 | 3 | 3 | 3 | 2 | 3 | 5 | 3 | 3 |
|  | DNNGP | 4 | 8 | 8 | 8 | 8 | 5 | 8 | 4 | 8 | 8 |
|  | SVR | 5 | 7 | 7 | 7 | 7 | 4 | 2 | 1 | 1 | 4 |
|  | BRR | 6 | 4 | 4 | 4 | 4 | 6 | 5 | 5 | 6 | 5 |
|  | BL | 7 | 6 | 6 | 6 | 6 | 8 | 7 | 5 | 5 | 6 |
|  | BayesA | 8 | 5 | 5 | 5 | 5 | 7 | 6 | 3 | 7 | 7 |

PH*,* plant height; PCC, Pearson’s correlation coefficient; MAE, mean absolute error; MSE, mean squared error; RMSE, root mean squared error; R-squared, coefficient of determination R²; SRCC, Spearman's rank correlation coefficient; NDCG@5, top 5 normalized discounted cumulative gain; THR@10%, top 10% hit rate; BHR@70%, bottom 70% hit rate; CICE, combined index for correlation and error. rrBLUP, ridge regression best linear unbiased prediction; BL, Bayesian LASSO; BRR, Bayesian ridge regression; LightGBM, light gradient boosting machine; SVR, support vector regression; RF, random forest; DNNGP, deep neural network for genomic prediction.

**Supplementary Table S3.** Score and ranking of eight prediction methods on PCC and nine other evaluation metrics for the trait FLW over 10-fold cross validation on the IRRI dataset.

|  | **Method** | **PCC** | **MAE** | **MSE** | **RMSE** | **R-squared** | **SRCC** | **NDCG@5** | **THR@10%** | **BHR@70%** | **CICE** |
| --- | --- | --- | --- | --- | --- | --- | --- | --- | --- | --- | --- |
| Score | LightGBM | 0.656 | 2.701 | 12.474 | 3.495 | 0.375 | 0.666 | 0.979 | 0.267 | 0.847 | 0.765 |
|  | RFR | 0.640 | 2.921 | 12.653 | 3.529 | 0.369 | 0.600 | 0.966 | 0.033 | 0.800 | 0.752 |
|  | rrBLUP | 0.581 | 2.965 | 14.679 | 3.792 | 0.281 | 0.563 | 0.976 | 0.300 | 0.826 | 0.721 |
|  | DNNGP | 0.520 | 18.870 | 529.374 | 22.807 | -25.615 | 0.515 | 0.964 | 0.400 | 0.732 | 0.454 |
|  | BRR | 0.440 | 3.382 | 17.578 | 4.165 | 0.145 | 0.443 | 0.970 | 0.267 | 0.816 | 0.642 |
|  | BL | 0.390 | 3.529 | 19.446 | 4.386 | 0.052 | 0.406 | 0.967 | 0.200 | 0.811 | 0.614 |
|  | SVR | 0.383 | 4.962 | 254.357 | 9.682 | -12.703 | 0.488 | 0.974 | 0.333 | 0.821 | 0.582 |
|  | BayesA | 0.378 | 3.554 | 19.898 | 4.431 | 0.032 | 0.392 | 0.965 | 0.233 | 0.816 | 0.607 |
| Ranking | LightGBM | 1 | 1 | 1 | 1 | 1 | 1 | 1 | 4 | 1 | 1 |
|  | RFR | 2 | 2 | 2 | 2 | 2 | 2 | 6 | 8 | 7 | 2 |
|  | rrBLUP | 3 | 3 | 3 | 3 | 3 | 3 | 2 | 3 | 2 | 3 |
|  | DNNGP | 4 | 8 | 8 | 8 | 8 | 4 | 8 | 1 | 8 | 8 |
|  | BRR | 5 | 4 | 4 | 4 | 4 | 6 | 4 | 4 | 5 | 4 |
|  | BL | 6 | 5 | 5 | 5 | 5 | 7 | 5 | 7 | 6 | 5 |
|  | SVR | 7 | 7 | 7 | 7 | 7 | 5 | 3 | 2 | 3 | 7 |
|  | BayesA | 8 | 6 | 6 | 6 | 6 | 8 | 7 | 6 | 4 | 6 |

FLW, flowering date; PCC, Pearson’s correlation coefficient; MAE, mean absolute error; MSE, mean squared error; RMSE, root mean squared error; R-squared, coefficient of determination R²; SRCC, Spearman's rank correlation coefficient; NDCG@5, top 5 normalized discounted cumulative gain; THR@10%, top 10% hit rate; BHR@70%, bottom 70% hit rate; CICE, combined index for correlation and error. rrBLUP, ridge regression best linear unbiased prediction; BL, Bayesian LASSO; BRR, Bayesian ridge regression; LightGBM, light gradient boosting machine; SVR, support vector regression; RF, random forest; DNNGP, deep neural network for genomic prediction.

**Supplementary Table S4.** Score and ranking of eight prediction methods on PCC and nine other evaluation metrics for the trait GrW over 10-fold cross validation on the IRRI dataset.

|  | **Method** | **PCC** | **MAE** | **MSE** | **RMSE** | **R-squared** | **SRCC** | **NDCG@5** | **THR@10%** | **BHR@70%** | **CICE** |
| --- | --- | --- | --- | --- | --- | --- | --- | --- | --- | --- | --- |
| Score | RFR | 0.361 | 0.067 | 0.008 | 0.087 | 0.081 | 0.309 | 0.959 | 0.233 | 0.737 | 0.635 |
|  | rrBLUP | 0.341 | 0.069 | 0.008 | 0.089 | 0.054 | 0.308 | 0.972 | 0.167 | 0.768 | 0.624 |
|  | LightGBM | 0.285 | 0.071 | 0.009 | 0.093 | -0.058 | 0.284 | 0.968 | 0.133 | 0.753 | 0.594 |
|  | DNNGP | 0.259 | 1.292 | 1.918 | 1.354 | -240.297 | 0.229 | 0.962 | 0.267 | 0.642 | 0.208 |
|  | SVR | 0.115 | 0.113 | 0.069 | 0.198 | -5.665 | 0.173 | 0.964 | 0.133 | 0.726 | 0.483 |
|  | BRR | 0.114 | 0.072 | 0.009 | 0.095 | -0.069 | 0.096 | 0.962 | 0.100 | 0.705 | 0.508 |
|  | BayesA | 0.104 | 0.077 | 0.010 | 0.100 | -0.203 | 0.082 | 0.961 | 0.100 | 0.700 | 0.500 |
|  | BL | 0.099 | 0.073 | 0.009 | 0.096 | -0.098 | 0.086 | 0.961 | 0.100 | 0.705 | 0.500 |
| Ranking | RFR | 1 | 1 | 1 | 1 | 1 | 1 | 8 | 2 | 3 | 1 |
|  | rrBLUP | 2 | 2 | 2 | 2 | 2 | 2 | 1 | 3 | 1 | 2 |
|  | LightGBM | 3 | 3 | 3 | 3 | 3 | 3 | 2 | 4 | 2 | 3 |
|  | DNNGP | 4 | 8 | 8 | 8 | 8 | 4 | 4 | 1 | 8 | 8 |
|  | SVR | 5 | 7 | 7 | 7 | 7 | 5 | 3 | 5 | 4 | 7 |
|  | BRR | 6 | 4 | 4 | 4 | 4 | 6 | 5 | 6 | 6 | 4 |
|  | BayesA | 7 | 6 | 6 | 6 | 6 | 8 | 7 | 6 | 7 | 6 |
|  | BL | 8 | 5 | 5 | 5 | 5 | 7 | 6 | 6 | 5 | 5 |

GrW, grain width; PCC, Pearson’s correlation coefficient; MAE, mean absolute error; MSE, mean squared error; RMSE, root mean squared error; R-squared, coefficient of determination R²; SRCC, Spearman's rank correlation coefficient; NDCG@5, top 5 normalized discounted cumulative gain; THR@10%, top 10% hit rate; BHR@70%, bottom 70% hit rate; CICE, combined index for correlation and error. rrBLUP, ridge regression best linear unbiased prediction; BL, Bayesian LASSO; BRR, Bayesian ridge regression; LightGBM, light gradient boosting machine; SVR, support vector regression; RF, random forest; DNNGP, deep neural network for genomic prediction.

**Supplementary Table S5.** Score and ranking of eight prediction methods on PCC and nine other evaluation metrics for the trait PedL over 10-fold cross validation on the IRRI dataset.

|  | **Method** | **PCC** | **MAE** | **MSE** | **RMSE** | **R-squared** | **SRCC** | **NDCG@5** | **THR@10%** | **BHR@70%** | **CICE** |
| --- | --- | --- | --- | --- | --- | --- | --- | --- | --- | --- | --- |
| Score | rrBLUP | 0.354 | 0.727 | 0.845 | 0.915 | 0.079 | 0.337 | 0.788 | 0.367 | 0.742 | 0.607 |
|  | DNNGP | 0.316 | 2.203 | 6.124 | 2.449 | -5.796 | 0.281 | 0.748 | 0.333 | 0.684 | 0.474 |
|  | RFR | 0.283 | 0.756 | 0.895 | 0.943 | 0.007 | 0.322 | 0.737 | 0.267 | 0.742 | 0.569 |
|  | LightGBM | 0.244 | 0.781 | 0.971 | 0.983 | -0.089 | 0.271 | 0.770 | 0.200 | 0.721 | 0.547 |
|  | SVR | 0.214 | 1.119 | 11.415 | 2.174 | -13.288 | 0.284 | 0.762 | 0.200 | 0.758 | 0.503 |
|  | BayesA | 0.016 | 0.841 | 1.157 | 1.069 | -0.258 | 0.051 | 0.722 | 0.133 | 0.705 | 0.428 |
|  | BRR | 0.008 | 0.800 | 1.033 | 1.009 | -0.115 | 0.041 | 0.707 | 0.167 | 0.700 | 0.427 |
|  | BL | 0.003 | 0.822 | 1.096 | 1.040 | -0.187 | 0.049 | 0.716 | 0.167 | 0.711 | 0.423 |
| Ranking | rrBLUP | 1 | 1 | 1 | 1 | 1 | 1 | 1 | 1 | 2 | 1 |
|  | DNNGP | 2 | 8 | 7 | 8 | 7 | 4 | 4 | 2 | 8 | 5 |
|  | RFR | 3 | 2 | 2 | 2 | 2 | 2 | 5 | 3 | 2 | 2 |
|  | LightGBM | 4 | 3 | 3 | 3 | 3 | 5 | 2 | 4 | 4 | 3 |
|  | SVR | 5 | 7 | 8 | 7 | 8 | 3 | 3 | 5 | 1 | 4 |
|  | BayesA | 6 | 6 | 6 | 6 | 6 | 6 | 6 | 8 | 6 | 6 |
|  | BRR | 7 | 4 | 4 | 4 | 4 | 8 | 8 | 6 | 7 | 7 |
|  | BL | 8 | 5 | 5 | 5 | 5 | 7 | 7 | 6 | 5 | 8 |

PedL, peduncle length; PCC, Pearson’s correlation coefficient; MAE, mean absolute error; MSE, mean squared error; RMSE, root mean squared error; R-squared, coefficient of determination R²; SRCC, Spearman's rank correlation coefficient; NDCG@5, top 5 normalized discounted cumulative gain; THR@10%, top 10% hit rate; BHR@70%, bottom 70% hit rate; CICE, combined index for correlation and error. rrBLUP, ridge regression best linear unbiased prediction; BL, Bayesian LASSO; BRR, Bayesian ridge regression; LightGBM, light gradient boosting machine; SVR, support vector regression; RF, random forest; DNNGP, deep neural network for genomic prediction.

**Supplementary Table S6.** Score and ranking of eight prediction methods on PCC and nine other evaluation metrics for the trait YLD over 10-fold cross validation on the IRRI dataset.

|  | **Method** | **PCC** | **MAE** | **MSE** | **RMSE** | **R-squared** | **SRCC** | **NDCG@5** | **THR@10%** | **BHR@70%** | **CICE** |
| --- | --- | --- | --- | --- | --- | --- | --- | --- | --- | --- | --- |
| Score | BRR | 0.578 | 519.057 | 456130.223 | 663.090 | 0.155 | 0.549 | 0.919 | 0.500 | 0.847 | 0.733 |
|  | BayesA | 0.470 | 526.934 | 465798.968 | 673.153 | 0.124 | 0.446 | 0.911 | 0.400 | 0.816 | 0.678 |
|  | DNNGP | 0.348 | 955.028 | 1446542.556 | 1179.533 | -1.856 | 0.319 | 0.884 | 0.200 | 0.679 | 0.575 |
|  | rrBLUP | 0.269 | 558.085 | 531127.499 | 716.423 | 0.033 | 0.255 | 0.865 | 0.200 | 0.763 | 0.574 |
|  | RFR | 0.267 | 562.378 | 521123.394 | 712.208 | 0.037 | 0.284 | 0.883 | 0.233 | 0.779 | 0.573 |
|  | LightGBM | 0.261 | 574.993 | 551736.438 | 733.518 | -0.054 | 0.254 | 0.866 | 0.167 | 0.763 | 0.569 |
|  | SVR | 0.158 | 976.525 | 8739848.314 | 1904.688 | -17.071 | 0.191 | 0.826 | 0.167 | 0.753 | 0.479 |
|  | BL | 0.083 | 654.469 | 712444.551 | 836.107 | -0.386 | 0.081 | 0.835 | 0.133 | 0.721 | 0.472 |
| Ranking | BRR | 1 | 1 | 1 | 1 | 1 | 1 | 1 | 1 | 1 | 1 |
|  | BayesA | 2 | 2 | 2 | 2 | 2 | 2 | 2 | 2 | 2 | 2 |
|  | DNNGP | 3 | 7 | 7 | 7 | 7 | 3 | 3 | 4 | 8 | 3 |
|  | rrBLUP | 4 | 3 | 4 | 4 | 4 | 5 | 6 | 4 | 5 | 4 |
|  | RFR | 5 | 4 | 3 | 3 | 3 | 4 | 4 | 3 | 3 | 5 |
|  | LightGBM | 6 | 5 | 5 | 5 | 5 | 6 | 5 | 6 | 4 | 6 |
|  | SVR | 7 | 8 | 8 | 8 | 8 | 7 | 8 | 7 | 6 | 7 |
|  | BL | 8 | 6 | 6 | 6 | 6 | 8 | 7 | 8 | 7 | 8 |

YLD, grain yield; PCC, Pearson’s correlation coefficient; MAE, mean absolute error; MSE, mean squared error; RMSE, root mean squared error; R-squared, coefficient of determination R²; SRCC, Spearman's rank correlation coefficient; NDCG@5, top 5 normalized discounted cumulative gain; THR@10%, top 10% hit rate; BHR@70%, bottom 70% hit rate; CICE, combined index for correlation and error. rrBLUP, ridge regression best linear unbiased prediction; BL, Bayesian LASSO; BRR, Bayesian ridge regression; LightGBM, light gradient boosting machine; SVR, support vector regression; RF, random forest; DNNGP, deep neural network for genomic prediction.

**Supplementary Table S7.** Score and ranking of eight prediction methods on PCC and nine other evaluation metrics for the trait Exs over 10-fold cross validation on the IRRI dataset.

|  | **Method** | **PCC** | **MAE** | **MSE** | **RMSE** | **R-squared** | **SRCC** | **NDCG@5** | **THR@10%** | **BHR@70%** | **CICE** |
| --- | --- | --- | --- | --- | --- | --- | --- | --- | --- | --- | --- |
| Score | DNNGP | 0.103 | 0.958 | 2.559 | 1.208 | -1974.745 | 0.129 | 0.934 | 0.167 | 0.679 | 0.072 |
|  | SVR | 0.023 | 0.055 | 0.018 | 0.098 | -12.051 | 0.011 | 0.928 | 0.067 | 0.700 | 0.428 |
|  | BL | 0.017 | 0.031 | 0.003 | 0.050 | -0.134 | 0.020 | 0.928 | 0.067 | 0.742 | 0.459 |
|  | BRR | 0.009 | 0.031 | 0.003 | 0.049 | -0.121 | -0.002 | 0.926 | 0.067 | 0.732 | 0.456 |
|  | BayesA | 0.003 | 0.032 | 0.003 | 0.052 | -0.244 | 0.007 | 0.927 | 0.100 | 0.737 | 0.451 |
|  | rrBLUP | 0.002 | 0.031 | 0.002 | 0.048 | -0.047 | 0.025 | 0.927 | 0.133 | 0.716 | 0.452 |
|  | RFR | -0.016 | 0.031 | 0.003 | 0.048 | -0.081 | 0.042 | 0.929 | 0.133 | 0.711 | 0.442 |
|  | LightGBM | -0.058 | 0.036 | 0.003 | 0.055 | -0.535 | -0.028 | 0.927 | 0.067 | 0.705 | 0.414 |
| Ranking | DNNGP | 1 | 8 | 8 | 8 | 8 | 1 | 1 | 1 | 8 | 8 |
|  | SVR | 2 | 7 | 7 | 7 | 7 | 5 | 3 | 5 | 7 | 6 |
|  | BL | 3 | 4 | 4 | 4 | 4 | 4 | 4 | 5 | 1 | 1 |
|  | BRR | 4 | 1 | 3 | 3 | 3 | 7 | 8 | 5 | 3 | 2 |
|  | BayesA | 5 | 5 | 5 | 5 | 5 | 6 | 5 | 4 | 2 | 4 |
|  | rrBLUP | 6 | 2 | 1 | 1 | 1 | 3 | 7 | 3 | 4 | 3 |
|  | RFR | 7 | 3 | 2 | 2 | 2 | 2 | 2 | 2 | 5 | 5 |
|  | LightGBM | 8 | 6 | 6 | 6 | 6 | 8 | 6 | 5 | 6 | 7 |

Exs, panicle exertion rate; PCC, Pearson’s correlation coefficient; MAE, mean absolute error; MSE, mean squared error; RMSE, root mean squared error; R-squared, coefficient of determination R²; SRCC, Spearman's rank correlation coefficient; NDCG@5, top 5 normalized discounted cumulative gain; THR@10%, top 10% hit rate; BHR@70%, bottom 70% hit rate; CICE, combined index for correlation and error. rrBLUP, ridge regression best linear unbiased prediction; BL, Bayesian LASSO; BRR, Bayesian ridge regression; LightGBM, light gradient boosting machine; SVR, support vector regression; RF, random forest; DNNGP, deep neural network for genomic prediction.

**Supplementary Table S8.** Score and ranking of eight prediction methods on PCC and nine other evaluation metrics for the trait Lg over 10-fold cross validation on the IRRI dataset.

|  | **Method** | **PCC** | **MAE** | **MSE** | **RMSE** | **R-squared** | **SRCC** | **NDCG@5** | **THR@10%** | **BHR@70%** | **CICE** |
| --- | --- | --- | --- | --- | --- | --- | --- | --- | --- | --- | --- |
| Score | DNNGP | 0.159 | 0.635 | 0.889 | 0.873 | -1.899 | 0.166 | 0.642 | 0.233 | 0.658 | 0.509 |
|  | rrBLUP | 0.106 | 0.328 | 0.392 | 0.572 | -0.282 | 0.094 | 0.613 | 0.233 | 0.716 | 0.516 |
|  | SVR | 0.050 | 0.629 | 4.656 | 1.325 | -10.657 | 0.065 | 0.633 | 0.167 | 0.726 | 0.455 |
|  | RFR | 0.007 | 0.329 | 0.394 | 0.575 | -0.376 | -0.006 | 0.622 | 0.200 | 0.726 | 0.466 |
|  | LightGBM | -0.010 | 0.383 | 0.469 | 0.645 | -0.941 | 0.022 | 0.575 | 0.133 | 0.700 | 0.451 |
|  | BRR | -0.052 | 0.351 | 0.431 | 0.605 | -0.715 | 0.003 | 0.572 | 0.067 | 0.716 | 0.434 |
|  | BL | -0.055 | 0.364 | 0.450 | 0.625 | -1.214 | -0.012 | 0.563 | 0.067 | 0.732 | 0.431 |
|  | BayesA | -0.061 | 0.372 | 0.493 | 0.657 | -2.010 | -0.042 | 0.575 | 0.067 | 0.721 | 0.427 |
| Ranking | DNNGP | 1 | 8 | 7 | 7 | 6 | 1 | 1 | 1 | 8 | 2 |
|  | rrBLUP | 2 | 1 | 1 | 1 | 1 | 2 | 4 | 1 | 5 | 1 |
|  | SVR | 3 | 7 | 8 | 8 | 8 | 3 | 2 | 4 | 2 | 4 |
|  | RFR | 4 | 2 | 2 | 2 | 2 | 6 | 3 | 3 | 2 | 3 |
|  | LightGBM | 5 | 6 | 5 | 5 | 4 | 4 | 6 | 5 | 7 | 5 |
|  | BRR | 6 | 3 | 3 | 3 | 3 | 5 | 7 | 6 | 6 | 6 |
|  | BL | 7 | 4 | 4 | 4 | 5 | 7 | 8 | 6 | 1 | 7 |
|  | BayesA | 8 | 5 | 6 | 6 | 7 | 8 | 5 | 6 | 4 | 8 |

Lg, lodging score; PCC, Pearson’s correlation coefficient; MAE, mean absolute error; MSE, mean squared error; RMSE, root mean squared error; R-squared, coefficient of determination R²; SRCC, Spearman's rank correlation coefficient; NDCG@5, top 5 normalized discounted cumulative gain; THR@10%, top 10% hit rate; BHR@70%, bottom 70% hit rate; CICE, combined index for correlation and error. rrBLUP, ridge regression best linear unbiased prediction; BL, Bayesian LASSO; BRR, Bayesian ridge regression; LightGBM, light gradient boosting machine; SVR, support vector regression; RF, random forest; DNNGP, deep neural network for genomic prediction.

**Supplementary Table S9.** Score and ranking of eight prediction methods on PCC and nine other evaluation metrics for the trait GY over 10-fold cross validation on the wheat599 dataset with 1279 markers (without dimensionality reduction).

|  | **Method** | **PCC** | **MAE** | **MSE** | **RMSE** | **R-squared** | **SRCC** | **NDCG@5** | **THR@10%** | **BHR@70%** | **CICE** |
| --- | --- | --- | --- | --- | --- | --- | --- | --- | --- | --- | --- |
| Score | BayesA | 0.727 | 0.605 | 0.551 | 0.740 | 0.426 | 0.676 | 0.680 | 0.450 | 0.849 | 0.813 |
|  | BL | 0.704 | 0.620 | 0.580 | 0.760 | 0.394 | 0.649 | 0.610 | 0.417 | 0.837 | 0.800 |
|  | RFR | 0.574 | 0.639 | 0.666 | 0.811 | 0.310 | 0.536 | 0.383 | 0.233 | 0.776 | 0.734 |
|  | BRR | 0.570 | 0.672 | 0.685 | 0.826 | 0.283 | 0.494 | 0.378 | 0.267 | 0.798 | 0.729 |
|  | LightGBM | 0.535 | 0.654 | 0.729 | 0.849 | 0.244 | 0.503 | 0.381 | 0.233 | 0.776 | 0.714 |
|  | DNNGP | 0.514 | 1.153 | 2.147 | 1.459 | -1.244 | 0.476 | 0.379 | 0.217 | 0.754 | 0.665 |
|  | SVR | 0.513 | 0.680 | 0.766 | 0.870 | 0.201 | 0.487 | 0.165 | 0.133 | 0.778 | 0.700 |
|  | rrBLUP | 0.511 | 0.674 | 0.728 | 0.850 | 0.241 | 0.471 | 0.264 | 0.167 | 0.771 | 0.700 |
| Ranking | BayesA | 1 | 1 | 1 | 1 | 1 | 1 | 1 | 1 | 1 | 1 |
|  | BL | 2 | 2 | 2 | 2 | 2 | 2 | 2 | 2 | 2 | 2 |
|  | RFR | 3 | 3 | 3 | 3 | 3 | 3 | 3 | 4 | 5 | 3 |
|  | BRR | 4 | 5 | 4 | 4 | 4 | 5 | 6 | 3 | 3 | 4 |
|  | LightGBM | 5 | 4 | 6 | 5 | 5 | 4 | 4 | 5 | 5 | 5 |
|  | DNNGP | 6 | 8 | 8 | 8 | 8 | 7 | 5 | 6 | 8 | 8 |
|  | SVR | 7 | 7 | 7 | 7 | 7 | 6 | 8 | 8 | 4 | 6 |
|  | rrBLUP | 8 | 6 | 5 | 6 | 6 | 8 | 7 | 7 | 7 | 7 |

GY*,* average grain yield; PCC, Pearson’s correlation coefficient; MAE, mean absolute error; MSE, mean squared error; RMSE, root mean squared error; R-squared, coefficient of determination R²; SRCC, Spearman's rank correlation coefficient; NDCG@5, top 5 normalized discounted cumulative gain; THR@10%, top 10% hit rate; BHR@70%, bottom 70% hit rate; CICE, combined index for correlation and error. rrBLUP, ridge regression best linear unbiased prediction; BL, Bayesian LASSO; BRR, Bayesian ridge regression; LightGBM, light gradient boosting machine; SVR, support vector regression; RF, random forest; DNNGP, deep neural network for genomic prediction.

**Supplementary Table S10.** Score and ranking of eight prediction methods on PCC and nine other evaluation metrics for the trait GY over 10-fold cross validation on the wheat599 dataset with 251 principal components after dimensionality reduction.

|  | **Method** | **PCC** | **MAE** | **MSE** | **RMSE** | **R-squared** | **SRCC** | **NDCG@5** | **THR@10%** | **BHR@70%** | **CICE** |
| --- | --- | --- | --- | --- | --- | --- | --- | --- | --- | --- | --- |
| Score | BayesA | 0.745 | 0.600 | 0.539 | 0.732 | 0.437 | 0.701 | 0.719 | 0.467 | 0.856 | 0.823 |
|  | BL | 0.712 | 0.620 | 0.579 | 0.759 | 0.396 | 0.657 | 0.664 | 0.400 | 0.841 | 0.805 |
|  | BRR | 0.575 | 0.672 | 0.684 | 0.826 | 0.284 | 0.502 | 0.424 | 0.267 | 0.793 | 0.732 |
|  | rrBLUP | 0.503 | 0.680 | 0.737 | 0.855 | 0.231 | 0.459 | 0.310 | 0.167 | 0.766 | 0.695 |
|  | LightGBM | 0.483 | 0.684 | 0.755 | 0.866 | 0.213 | 0.452 | 0.444 | 0.250 | 0.776 | 0.685 |
|  | DNNGP | 0.479 | 0.690 | 0.777 | 0.878 | 0.189 | 0.454 | 0.304 | 0.183 | 0.754 | 0.683 |
|  | SVR | 0.410 | 0.725 | 0.830 | 0.908 | 0.133 | 0.398 | 0.237 | 0.133 | 0.749 | 0.645 |
|  | RFR | 0.378 | 0.777 | 0.933 | 0.963 | 0.031 | 0.345 | 0.315 | 0.133 | 0.761 | 0.625 |
| Ranking | BayesA | 1 | 1 | 1 | 1 | 1 | 1 | 1 | 1 | 1 | 1 |
|  | BL | 2 | 2 | 2 | 2 | 2 | 2 | 2 | 2 | 2 | 2 |
|  | BRR | 3 | 3 | 3 | 3 | 3 | 3 | 4 | 3 | 3 | 3 |
|  | rrBLUP | 4 | 4 | 4 | 4 | 4 | 4 | 6 | 6 | 5 | 4 |
|  | LightGBM | 5 | 5 | 5 | 5 | 5 | 6 | 3 | 4 | 4 | 5 |
|  | DNNGP | 6 | 6 | 6 | 6 | 6 | 5 | 7 | 5 | 7 | 6 |
|  | SVR | 7 | 7 | 7 | 7 | 7 | 7 | 8 | 7 | 8 | 7 |
|  | RFR | 8 | 8 | 8 | 8 | 8 | 8 | 5 | 7 | 6 | 8 |

GY*,* average grain yield; PCC, Pearson’s correlation coefficient; MAE, mean absolute error; MSE, mean squared error; RMSE, root mean squared error; R-squared, coefficient of determination R²; SRCC, Spearman's rank correlation coefficient; NDCG@5, top 5 normalized discounted cumulative gain; THR@10%, top 10% hit rate; BHR@70%, bottom 70% hit rate; CICE, combined index for correlation and error. rrBLUP, ridge regression best linear unbiased prediction; BL, Bayesian LASSO; BRR, Bayesian ridge regression; LightGBM, light gradient boosting machine; SVR, support vector regression; RF, random forest; DNNGP, deep neural network for genomic prediction.

**Supplementary Table S11.** Score and ranking of eight prediction methods on PCC and nine other evaluation metrics for the trait Biomass over 10-fold cross validation on the wheat487 dataset.

|  | **Method** | **PCC** | **MAE** | **MSE** | **RMSE** | **R-squared** | **SRCC** | **NDCG@5** | **THR@10%** | **BHR@70%** | **CICE** |
| --- | --- | --- | --- | --- | --- | --- | --- | --- | --- | --- | --- |
| Score | rrBLUP | 0.697 | 0.026 | 0.001 | 0.035 | 0.461 | 0.687 | 0.869 | 0.220 | 0.841 | 0.797 |
|  | RFR | 0.691 | 0.027 | 0.001 | 0.035 | 0.458 | 0.681 | 0.866 | 0.200 | 0.832 | 0.793 |
|  | LightGBM | 0.682 | 0.027 | 0.001 | 0.035 | 0.435 | 0.679 | 0.882 | 0.180 | 0.818 | 0.789 |
|  | DNNGP | 0.648 | 0.188 | 0.078 | 0.236 | -35.742 | 0.652 | 0.877 | 0.240 | 0.797 | 0.555 |
|  | SVR | 0.626 | 0.030 | 0.002 | 0.039 | 0.308 | 0.632 | 0.861 | 0.220 | 0.826 | 0.756 |
|  | BRR | 0.214 | 0.039 | 0.002 | 0.047 | -0.001 | 0.216 | 0.787 | 0.140 | 0.747 | 0.532 |
|  | BL | 0.209 | 0.039 | 0.002 | 0.047 | -0.001 | 0.220 | 0.783 | 0.120 | 0.756 | 0.530 |
|  | BayesA | 0.143 | 0.041 | 0.003 | 0.050 | -0.124 | 0.145 | 0.763 | 0.140 | 0.724 | 0.493 |
| Ranking | rrBLUP | 1 | 1 | 2 | 1 | 1 | 1 | 3 | 2 | 1 | 1 |
|  | RFR | 2 | 2 | 1 | 2 | 2 | 2 | 4 | 4 | 2 | 2 |
|  | LightGBM | 3 | 3 | 3 | 3 | 3 | 3 | 1 | 5 | 4 | 3 |
|  | DNNGP | 4 | 8 | 8 | 8 | 8 | 4 | 2 | 1 | 5 | 5 |
|  | SVR | 5 | 4 | 4 | 4 | 4 | 5 | 5 | 2 | 3 | 4 |
|  | BRR | 6 | 5 | 5 | 6 | 6 | 7 | 6 | 6 | 7 | 6 |
|  | BL | 7 | 6 | 5 | 5 | 5 | 6 | 7 | 8 | 6 | 7 |
|  | BayesA | 8 | 7 | 7 | 7 | 7 | 8 | 8 | 6 | 8 | 8 |

PCC, Pearson’s correlation coefficient; MAE, mean absolute error; MSE, mean squared error; RMSE, root mean squared error; R-squared, coefficient of determination R²; SRCC, Spearman's rank correlation coefficient; NDCG@5, top 5 normalized discounted cumulative gain; THR@10%, top 10% hit rate; BHR@70%, bottom 70% hit rate; CICE, combined index for correlation and error. rrBLUP, ridge regression best linear unbiased prediction; BL, Bayesian LASSO; BRR, Bayesian ridge regression; LightGBM, light gradient boosting machine; SVR, support vector regression; RF, random forest; DNNGP, deep neural network for genomic prediction.

**Supplementary Table S12.** Score and ranking of eight prediction methods on PCC and nine other evaluation metrics for the trait Yield over 10-fold cross validation on the wheat487 dataset.

|  | **Method** | **PCC** | **MAE** | **MSE** | **RMSE** | **R-squared** | **SRCC** | **NDCG@5** | **THR@10%** | **BHR@70%** | **CICE** |
| --- | --- | --- | --- | --- | --- | --- | --- | --- | --- | --- | --- |
| Score | RFR | 0.786 | 0.321 | 0.171 | 0.412 | 0.602 | 0.771 | 0.906 | 0.340 | 0.847 | 0.840 |
|  | LightGBM | 0.783 | 0.311 | 0.171 | 0.411 | 0.601 | 0.771 | 0.886 | 0.280 | 0.847 | 0.840 |
|  | rrBLUP | 0.782 | 0.313 | 0.172 | 0.413 | 0.598 | 0.762 | 0.897 | 0.360 | 0.850 | 0.839 |
|  | DNNGP | 0.758 | 0.624 | 1.251 | 0.790 | -1.513 | 0.739 | 0.874 | 0.360 | 0.812 | 0.780 |
|  | SVR | 0.755 | 0.326 | 0.193 | 0.438 | 0.547 | 0.751 | 0.903 | 0.320 | 0.862 | 0.824 |
|  | BRR | 0.279 | 0.537 | 0.413 | 0.640 | 0.052 | 0.275 | 0.759 | 0.080 | 0.765 | 0.553 |
|  | BL | 0.249 | 0.542 | 0.424 | 0.649 | 0.027 | 0.248 | 0.765 | 0.120 | 0.762 | 0.538 |
|  | BayesA | 0.180 | 0.568 | 0.473 | 0.686 | -0.090 | 0.178 | 0.743 | 0.100 | 0.738 | 0.499 |
| Ranking | RFR | 1 | 3 | 2 | 2 | 1 | 1 | 1 | 3 | 4 | 2 |
|  | LightGBM | 2 | 1 | 1 | 1 | 2 | 2 | 4 | 5 | 3 | 1 |
|  | rrBLUP | 3 | 2 | 3 | 3 | 3 | 3 | 3 | 1 | 2 | 3 |
|  | DNNGP | 4 | 8 | 8 | 8 | 8 | 5 | 5 | 1 | 5 | 5 |
|  | SVR | 5 | 4 | 4 | 4 | 4 | 4 | 2 | 4 | 1 | 4 |
|  | BRR | 6 | 5 | 5 | 5 | 5 | 6 | 7 | 8 | 6 | 6 |
|  | BL | 7 | 6 | 6 | 6 | 6 | 7 | 6 | 6 | 7 | 7 |
|  | BayesA | 8 | 7 | 7 | 7 | 7 | 8 | 8 | 7 | 8 | 8 |

PCC, Pearson’s correlation coefficient; MAE, mean absolute error; MSE, mean squared error; RMSE, root mean squared error; R-squared, coefficient of determination R²; SRCC, Spearman's rank correlation coefficient; NDCG@5, top 5 normalized discounted cumulative gain; THR@10%, top 10% hit rate; BHR@70%, bottom 70% hit rate; CICE, combined index for correlation and error. rrBLUP, ridge regression best linear unbiased prediction; BL, Bayesian LASSO; BRR, Bayesian ridge regression; LightGBM, light gradient boosting machine; SVR, support vector regression; RF, random forest; DNNGP, deep neural network for genomic prediction.

**Supplementary Table S13.** Score and ranking of eight prediction methods on PCC and nine other evaluation metrics for the trait PH over 10-fold cross validation on the G2F_2017 dataset.

|  | **Method** | **PCC** | **MAE** | **MSE** | **RMSE** | **R-squared** | **SRCC** | **NDCG@5** | **THR@10%** | **BHR@70%** | **CICE** |
| --- | --- | --- | --- | --- | --- | --- | --- | --- | --- | --- | --- |
| Score | rrBLUP | 0.886 | 5.105 | 43.778 | 6.587 | 0.772 | 0.866 | 0.990 | 0.700 | 0.875 | 0.914 |
|  | LightGBM | 0.866 | 5.497 | 50.733 | 7.089 | 0.739 | 0.834 | 0.984 | 0.700 | 0.862 | 0.902 |
|  | SVR | 0.864 | 5.673 | 53.235 | 7.251 | 0.726 | 0.836 | 0.980 | 0.675 | 0.871 | 0.900 |
|  | DNNGP | 0.849 | 30.954 | 1502.262 | 38.113 | -6.586 | 0.818 | 0.987 | 0.650 | 0.808 | 0.773 |
|  | RFR | 0.677 | 9.135 | 128.234 | 11.253 | 0.351 | 0.733 | 0.966 | 0.425 | 0.846 | 0.788 |
|  | BRR | 0.533 | 10.246 | 160.452 | 12.576 | 0.190 | 0.544 | 0.954 | 0.450 | 0.792 | 0.710 |
|  | BayesA | 0.442 | 10.419 | 167.319 | 12.828 | 0.153 | 0.456 | 0.940 | 0.450 | 0.775 | 0.664 |
|  | BL | 0.397 | 10.675 | 175.020 | 13.143 | 0.111 | 0.399 | 0.938 | 0.400 | 0.771 | 0.640 |
| Ranking | rrBLUP | 1 | 1 | 1 | 1 | 1 | 1 | 1 | 1 | 1 | 1 |
|  | LightGBM | 2 | 2 | 2 | 2 | 2 | 3 | 3 | 1 | 3 | 2 |
|  | SVR | 3 | 3 | 3 | 3 | 3 | 2 | 4 | 3 | 2 | 3 |
|  | DNNGP | 4 | 8 | 8 | 8 | 8 | 4 | 2 | 4 | 5 | 5 |
|  | RFR | 5 | 4 | 4 | 4 | 4 | 5 | 5 | 7 | 4 | 4 |
|  | BRR | 6 | 5 | 5 | 5 | 5 | 6 | 6 | 5 | 6 | 6 |
|  | BayesA | 7 | 6 | 6 | 6 | 6 | 7 | 7 | 5 | 7 | 7 |
|  | BL | 8 | 7 | 7 | 7 | 7 | 8 | 8 | 8 | 8 | 8 |

PH, plant height; PCC, Pearson’s correlation coefficient; MAE, mean absolute error; MSE, mean squared error; RMSE, root mean squared error; R-squared, coefficient of determination R²; SRCC, Spearman's rank correlation coefficient; NDCG@5, top 5 normalized discounted cumulative gain; THR@10%, top 10% hit rate; BHR@70%, bottom 70% hit rate; CICE, combined index for correlation and error. rrBLUP, ridge regression best linear unbiased prediction; BL, Bayesian LASSO; BRR, Bayesian ridge regression; LightGBM, light gradient boosting machine; SVR, support vector regression; RF, random forest; DNNGP, deep neural network for genomic prediction.

**Supplementary Table S14.** Score and ranking of eight prediction methods on PCC and nine other evaluation metrics for the trait Yield over 10-fold cross validation on the G2F_2017 dataset.

|  | **Method** | **PCC** | **MAE** | **MSE** | **RMSE** | **R-squared** | **SRCC** | **NDCG@5** | **THR@10%** | **BHR@70%** | **CICE** |
| --- | --- | --- | --- | --- | --- | --- | --- | --- | --- | --- | --- |
| Score | DNNGP | 0.652 | 24.537 | 980.916 | 30.866 | -4.661 | 0.661 | 0.966 | 0.475 | 0.792 | 0.718 |
|  | rrBLUP | 0.650 | 7.638 | 108.879 | 10.291 | 0.394 | 0.661 | 0.974 | 0.625 | 0.863 | 0.789 |
|  | LightGBM | 0.642 | 7.790 | 113.711 | 10.530 | 0.367 | 0.653 | 0.972 | 0.550 | 0.833 | 0.784 |
|  | SVR | 0.626 | 7.825 | 116.052 | 10.621 | 0.358 | 0.611 | 0.964 | 0.525 | 0.846 | 0.776 |
|  | RFR | 0.482 | 9.390 | 154.943 | 12.341 | 0.135 | 0.499 | 0.954 | 0.425 | 0.804 | 0.696 |
|  | BRR | 0.151 | 10.278 | 184.175 | 13.478 | -0.034 | 0.173 | 0.894 | 0.125 | 0.746 | 0.527 |
|  | BL | 0.128 | 10.756 | 202.097 | 14.153 | -0.156 | 0.153 | 0.874 | 0.125 | 0.729 | 0.513 |
|  | BayesA | 0.127 | 10.523 | 193.147 | 13.828 | -0.096 | 0.152 | 0.879 | 0.150 | 0.738 | 0.514 |
| Ranking | DNNGP | 1 | 8 | 8 | 8 | 8 | 2 | 3 | 4 | 5 | 4 |
|  | rrBLUP | 2 | 1 | 1 | 1 | 1 | 1 | 1 | 1 | 1 | 1 |
|  | LightGBM | 3 | 2 | 2 | 2 | 2 | 3 | 2 | 2 | 3 | 2 |
|  | SVR | 4 | 3 | 3 | 3 | 3 | 4 | 4 | 3 | 2 | 3 |
|  | RFR | 5 | 4 | 4 | 4 | 4 | 5 | 5 | 5 | 4 | 5 |
|  | BRR | 6 | 5 | 5 | 5 | 5 | 6 | 6 | 7 | 6 | 6 |
|  | BL | 7 | 7 | 7 | 7 | 7 | 7 | 8 | 7 | 8 | 8 |
|  | BayesA | 8 | 6 | 6 | 6 | 6 | 8 | 7 | 6 | 7 | 7 |

PCC, Pearson’s correlation coefficient; MAE, mean absolute error; MSE, mean squared error; RMSE, root mean squared error; R-squared, coefficient of determination R²; SRCC, Spearman's rank correlation coefficient; NDCG@5, top 5 normalized discounted cumulative gain; THR@10%, top 10% hit rate; BHR@70%, bottom 70% hit rate; CICE, combined index for correlation and error. rrBLUP, ridge regression best linear unbiased prediction; BL, Bayesian LASSO; BRR, Bayesian ridge regression; LightGBM, light gradient boosting machine; SVR, support vector regression; RF, random forest; DNNGP, deep neural network for genomic prediction.

**Supplementary Table S15.** Score and ranking of eight prediction methods on PCC and nine other evaluation metrics for the trait DTP over 10-fold cross validation on the CNGWAS dataset.

|  | **Method** | **PCC** | **MAE** | **MSE** | **RMSE** | **R-squared** | **SRCC** | **NDCG@5** | **THR@10%** | **BHR@70%** | **CICE** |
| --- | --- | --- | --- | --- | --- | --- | --- | --- | --- | --- | --- |
| Score | rrBLUP | 0.680 | 2.111 | 7.508 | 2.731 | 0.444 | 0.655 | 0.966 | 0.380 | 0.851 | 0.799 |
|  | SVR | 0.679 | 2.112 | 7.505 | 2.730 | 0.444 | 0.655 | 0.967 | 0.380 | 0.849 | 0.798 |
|  | DNNGP | 0.664 | 11.511 | 202.408 | 14.067 | -14.688 | 0.649 | 0.967 | 0.440 | 0.826 | 0.645 |
|  | LightGBM | 0.638 | 2.238 | 8.369 | 2.883 | 0.375 | 0.604 | 0.970 | 0.440 | 0.843 | 0.776 |
|  | RFR | 0.546 | 2.655 | 11.629 | 3.395 | 0.147 | 0.540 | 0.957 | 0.360 | 0.834 | 0.722 |
|  | BRR | 0.314 | 2.883 | 13.178 | 3.607 | 0.038 | 0.334 | 0.926 | 0.200 | 0.774 | 0.602 |
|  | BL | 0.270 | 2.993 | 14.166 | 3.742 | -0.035 | 0.284 | 0.923 | 0.180 | 0.766 | 0.578 |
|  | BayesA | 0.251 | 3.088 | 15.125 | 3.864 | -0.104 | 0.250 | 0.924 | 0.180 | 0.754 | 0.567 |
| Ranking | rrBLUP | 1 | 1 | 2 | 2 | 2 | 2 | 4 | 3 | 1 | 1 |
|  | SVR | 2 | 2 | 1 | 1 | 1 | 1 | 2 | 3 | 2 | 2 |
|  | DNNGP | 3 | 8 | 8 | 8 | 8 | 3 | 3 | 1 | 5 | 5 |
|  | LightGBM | 4 | 3 | 3 | 3 | 3 | 4 | 1 | 1 | 3 | 3 |
|  | RFR | 5 | 4 | 4 | 4 | 4 | 5 | 5 | 5 | 4 | 4 |
|  | BRR | 6 | 5 | 5 | 5 | 5 | 6 | 6 | 6 | 6 | 6 |
|  | BL | 7 | 6 | 6 | 6 | 6 | 7 | 8 | 7 | 7 | 7 |
|  | BayesA | 8 | 7 | 7 | 7 | 7 | 8 | 7 | 7 | 8 | 8 |

DTP, days to pollen; PCC, Pearson’s correlation coefficient; MAE, mean absolute error; MSE, mean squared error; RMSE, root mean squared error; R-squared, coefficient of determination R²; SRCC, Spearman's rank correlation coefficient; NDCG@5, top 5 normalized discounted cumulative gain; THR@10%, top 10% hit rate; BHR@70%, bottom 70% hit rate; CICE, combined index for correlation and error. rrBLUP, ridge regression best linear unbiased prediction; BL, Bayesian LASSO; BRR, Bayesian ridge regression; LightGBM, light gradient boosting machine; SVR, support vector regression; RF, random forest; DNNGP, deep neural network for genomic prediction.

**Supplementary Table S16.** Score and ranking of eight prediction methods on PCC and nine other evaluation metrics for the trait PH over 10-fold cross validation on the CNGWAS dataset.

|  | **Method** | **PCC** | **MAE** | **MSE** | **RMSE** | **R-squared** | **SRCC** | **NDCG@5** | **THR@10%** | **BHR@70%** | **CICE** |
| --- | --- | --- | --- | --- | --- | --- | --- | --- | --- | --- | --- |
| Score | rrBLUP | 0.598 | 13.670 | 301.691 | 17.290 | 0.346 | 0.595 | 0.931 | 0.340 | 0.846 | 0.742 |
|  | SVR | 0.586 | 13.860 | 307.680 | 17.458 | 0.333 | 0.587 | 0.930 | 0.300 | 0.846 | 0.735 |
|  | DNNGP | 0.575 | 32.310 | 1607.669 | 40.023 | -2.526 | 0.574 | 0.933 | 0.320 | 0.809 | 0.664 |
|  | LightGBM | 0.533 | 14.626 | 334.957 | 18.232 | 0.272 | 0.521 | 0.929 | 0.300 | 0.817 | 0.706 |
|  | RFR | 0.394 | 16.994 | 435.263 | 20.819 | 0.058 | 0.392 | 0.917 | 0.300 | 0.791 | 0.627 |
|  | BRR | 0.275 | 17.056 | 451.161 | 21.142 | 0.025 | 0.257 | 0.900 | 0.180 | 0.757 | 0.568 |
|  | BayesA | 0.215 | 18.304 | 523.045 | 22.739 | -0.131 | 0.200 | 0.885 | 0.160 | 0.743 | 0.533 |
|  | BL | 0.189 | 18.654 | 543.842 | 23.163 | -0.173 | 0.178 | 0.874 | 0.160 | 0.743 | 0.519 |
| Ranking | rrBLUP | 1 | 1 | 1 | 1 | 1 | 1 | 2 | 1 | 1 | 1 |
|  | SVR | 2 | 2 | 2 | 2 | 2 | 2 | 3 | 3 | 2 | 2 |
|  | DNNGP | 3 | 8 | 8 | 8 | 8 | 3 | 1 | 2 | 4 | 4 |
|  | LightGBM | 4 | 3 | 3 | 3 | 3 | 4 | 4 | 3 | 3 | 3 |
|  | RFR | 5 | 4 | 4 | 4 | 4 | 5 | 5 | 3 | 5 | 5 |
|  | BRR | 6 | 5 | 5 | 5 | 5 | 6 | 6 | 6 | 6 | 6 |
|  | BayesA | 7 | 6 | 6 | 6 | 6 | 7 | 7 | 7 | 7 | 7 |
|  | BL | 8 | 7 | 7 | 7 | 7 | 8 | 8 | 7 | 7 | 8 |

PH, plant height; PCC, Pearson’s correlation coefficient; MAE, mean absolute error; MSE, mean squared error; RMSE, root mean squared error; R-squared, coefficient of determination R²; SRCC, Spearman's rank correlation coefficient; NDCG@5, top 5 normalized discounted cumulative gain; THR@10%, top 10% hit rate; BHR@70%, bottom 70% hit rate; CICE, combined index for correlation and error. rrBLUP, ridge regression best linear unbiased prediction; BL, Bayesian LASSO; BRR, Bayesian ridge regression; LightGBM, light gradient boosting machine; SVR, support vector regression; RF, random forest; DNNGP, deep neural network for genomic prediction.

**Supplementary Table S17.** Score and ranking of eight prediction methods on PCC and nine other evaluation metrics for the trait GW100 over 10-fold cross validation on the USNAM dataset.

|  | **Method** | **PCC** | **MAE** | **MSE** | **RMSE** | **R-squared** | **SRCC** | **NDCG@5** | **THR@10%** | **BHR@70%** | **CICE** |
| --- | --- | --- | --- | --- | --- | --- | --- | --- | --- | --- | --- |
| Score | rrBLUP | 0.709 | 1.598 | 4.137 | 2.033 | 0.498 | 0.697 | 0.909 | 0.480 | 0.842 | 0.816 |
|  | DNNGP | 0.680 | 3.355 | 17.968 | 4.216 | -1.195 | 0.670 | 0.894 | 0.436 | 0.829 | 0.763 |
|  | LightGBM | 0.663 | 1.694 | 4.656 | 2.156 | 0.435 | 0.651 | 0.886 | 0.442 | 0.827 | 0.791 |
|  | SVR | 0.618 | 1.919 | 5.938 | 2.436 | 0.276 | 0.603 | 0.893 | 0.413 | 0.817 | 0.763 |
|  | RFR | 0.460 | 2.144 | 7.316 | 2.702 | 0.115 | 0.461 | 0.824 | 0.273 | 0.772 | 0.679 |
|  | BRR | 0.142 | 2.332 | 8.594 | 2.928 | -0.039 | 0.149 | 0.738 | 0.160 | 0.723 | 0.516 |
|  | BL | 0.134 | 2.348 | 8.717 | 2.949 | -0.054 | 0.139 | 0.736 | 0.162 | 0.718 | 0.512 |
|  | BayesA | 0.123 | 2.374 | 8.896 | 2.979 | -0.076 | 0.125 | 0.733 | 0.164 | 0.719 | 0.506 |
| Ranking | rrBLUP | 1 | 1 | 1 | 1 | 1 | 1 | 1 | 1 | 1 | 1 |
|  | DNNGP | 2 | 8 | 8 | 8 | 8 | 2 | 2 | 3 | 2 | 4 |
|  | LightGBM | 3 | 2 | 2 | 2 | 2 | 3 | 4 | 2 | 3 | 2 |
|  | SVR | 4 | 3 | 3 | 3 | 3 | 4 | 3 | 4 | 4 | 3 |
|  | RFR | 5 | 4 | 4 | 4 | 4 | 5 | 5 | 5 | 5 | 5 |
|  | BRR | 6 | 5 | 5 | 5 | 5 | 6 | 6 | 8 | 6 | 6 |
|  | BL | 7 | 6 | 6 | 6 | 6 | 7 | 7 | 7 | 8 | 7 |
|  | BayesA | 8 | 7 | 7 | 7 | 7 | 8 | 8 | 6 | 7 | 8 |

GW100, 100 grain weight; PCC, Pearson’s correlation coefficient; MAE, mean absolute error; MSE, mean squared error; RMSE, root mean squared error; R-squared, coefficient of determination R²; SRCC, Spearman's rank correlation coefficient; NDCG@5, top 5 normalized discounted cumulative gain; THR@10%, top 10% hit rate; BHR@70%, bottom 70% hit rate; CICE, combined index for correlation and error. rrBLUP, ridge regression best linear unbiased prediction; BL, Bayesian LASSO; BRR, Bayesian ridge regression; LightGBM, light gradient boosting machine; SVR, support vector regression; RF, random forest; DNNGP, deep neural network for genomic prediction.

**Supplementary Table S18.** Score and ranking of eight prediction methods on PCC and nine other evaluation metrics for the trait PH over 10-fold cross validation on the USNAM dataset.

|  | **Method** | **PCC** | **MAE** | **MSE** | **RMSE** | **R-squared** | **SRCC** | **NDCG@5** | **THR@10%** | **BHR@70%** | **CICE** |
| --- | --- | --- | --- | --- | --- | --- | --- | --- | --- | --- | --- |
| Score | rrBLUP | 0.789 | 7.855 | 100.748 | 10.032 | 0.621 | 0.782 | 0.947 | 0.522 | 0.864 | 0.865 |
|  | DNNGP | 0.768 | 27.437 | 1218.153 | 34.853 | -3.587 | 0.760 | 0.945 | 0.500 | 0.854 | 0.787 |
|  | SVR | 0.743 | 8.890 | 128.546 | 11.335 | 0.516 | 0.733 | 0.938 | 0.489 | 0.850 | 0.838 |
|  | LightGBM | 0.742 | 8.626 | 120.264 | 10.954 | 0.549 | 0.732 | 0.938 | 0.493 | 0.848 | 0.838 |
|  | RFR | 0.398 | 12.234 | 234.529 | 15.307 | 0.119 | 0.458 | 0.893 | 0.316 | 0.772 | 0.653 |
|  | BRR | 0.372 | 12.135 | 230.247 | 15.159 | 0.136 | 0.329 | 0.822 | 0.198 | 0.753 | 0.640 |
|  | BL | 0.337 | 12.336 | 238.557 | 15.429 | 0.105 | 0.293 | 0.817 | 0.182 | 0.743 | 0.623 |
|  | BayesA | 0.331 | 12.381 | 240.727 | 15.503 | 0.096 | 0.288 | 0.824 | 0.187 | 0.743 | 0.619 |
| Ranking | rrBLUP | 1 | 1 | 1 | 1 | 1 | 1 | 1 | 1 | 1 | 1 |
|  | DNNGP | 2 | 8 | 8 | 8 | 8 | 2 | 2 | 2 | 2 | 4 |
|  | SVR | 3 | 3 | 3 | 3 | 3 | 3 | 3 | 4 | 3 | 3 |
|  | LightGBM | 4 | 2 | 2 | 2 | 2 | 4 | 4 | 3 | 4 | 2 |
|  | RFR | 5 | 5 | 5 | 5 | 5 | 5 | 5 | 5 | 5 | 5 |
|  | BRR | 6 | 4 | 4 | 4 | 4 | 6 | 7 | 6 | 6 | 6 |
|  | BL | 7 | 6 | 6 | 6 | 6 | 7 | 8 | 8 | 7 | 7 |
|  | BayesA | 8 | 7 | 7 | 7 | 7 | 8 | 6 | 7 | 8 | 8 |

PH, plant height; PCC, Pearson’s correlation coefficient; MAE, mean absolute error; MSE, mean squared error; RMSE, root mean squared error; R-squared, coefficient of determination R²; SRCC, Spearman's rank correlation coefficient; NDCG@5, top 5 normalized discounted cumulative gain; THR@10%, top 10% hit rate; BHR@70%, bottom 70% hit rate; CICE, combined index for correlation and error. rrBLUP, ridge regression best linear unbiased prediction; BL, Bayesian LASSO; BRR, Bayesian ridge regression; LightGBM, light gradient boosting machine; SVR, support vector regression; RF, random forest; DNNGP, deep neural network for genomic prediction.

**Supplementary Table S19.** Score and ranking of eight prediction methods on PCC and nine other evaluation metrics for the trait MSH over 10-fold cross validation on the millet827 dataset.

|  | **Method** | **PCC** | **MAE** | **MSE** | **RMSE** | **R-squared** | **SRCC** | **NDCG@5** | **THR@10%** | **BHR@70%** | **CICE** |
| --- | --- | --- | --- | --- | --- | --- | --- | --- | --- | --- | --- |
| Score | BRR | 0.459 | 10.179 | 168.191 | 12.943 | 0.188 | 0.429 | 0.872 | 0.325 | 0.786 | 0.682 |
|  | LightGBM | 0.358 | 10.838 | 186.507 | 13.631 | 0.101 | 0.295 | 0.880 | 0.325 | 0.744 | 0.628 |
|  | rrBLUP | 0.353 | 10.914 | 184.419 | 13.545 | 0.112 | 0.293 | 0.883 | 0.350 | 0.760 | 0.625 |
|  | BayesA | 0.338 | 10.867 | 192.118 | 13.836 | 0.070 | 0.315 | 0.856 | 0.250 | 0.768 | 0.618 |
|  | SVR | 0.337 | 11.598 | 209.959 | 14.442 | -0.012 | 0.276 | 0.880 | 0.325 | 0.747 | 0.614 |
|  | BL | 0.332 | 10.953 | 196.909 | 14.003 | 0.048 | 0.312 | 0.850 | 0.300 | 0.763 | 0.614 |
|  | DNNGP | 0.324 | 21.782 | 736.822 | 27.040 | -2.569 | 0.271 | 0.867 | 0.350 | 0.735 | 0.565 |
|  | RFR | 0.243 | 11.249 | 202.175 | 14.201 | 0.025 | 0.171 | 0.850 | 0.338 | 0.742 | 0.569 |
| Ranking | BRR | 1 | 1 | 1 | 1 | 1 | 1 | 4 | 4 | 1 | 1 |
|  | LightGBM | 2 | 2 | 3 | 3 | 3 | 4 | 2 | 4 | 6 | 2 |
|  | rrBLUP | 3 | 4 | 2 | 2 | 2 | 5 | 1 | 1 | 4 | 3 |
|  | BayesA | 4 | 3 | 4 | 4 | 4 | 2 | 6 | 8 | 2 | 4 |
|  | SVR | 5 | 7 | 7 | 7 | 7 | 6 | 3 | 4 | 5 | 5 |
|  | BL | 6 | 5 | 5 | 5 | 5 | 3 | 8 | 7 | 3 | 6 |
|  | DNNGP | 7 | 8 | 8 | 8 | 8 | 7 | 5 | 1 | 8 | 8 |
|  | RFR | 8 | 6 | 6 | 6 | 6 | 8 | 7 | 3 | 7 | 7 |

MSH, main stem height; PCC, Pearson’s correlation coefficient; MAE, mean absolute error; MSE, mean squared error; RMSE, root mean squared error; R-squared, coefficient of determination R²; SRCC, Spearman's rank correlation coefficient; NDCG@5, top 5 normalized discounted cumulative gain; THR@10%, top 10% hit rate; BHR@70%, bottom 70% hit rate; CICE, combined index for correlation and error. rrBLUP, ridge regression best linear unbiased prediction; BL, Bayesian LASSO; BRR, Bayesian ridge regression; LightGBM, light gradient boosting machine; SVR, support vector regression; RF, random forest; DNNGP, deep neural network for genomic prediction.

**Supplementary Table S20.** Score and ranking of eight prediction methods on PCC and nine other evaluation metrics for the trait PGW over 10-fold cross validation on the millet827 dataset.

|  | **Method** | **PCC** | **MAE** | **MSE** | **RMSE** | **R-squared** | **SRCC** | **NDCG@5** | **THR@10%** | **BHR@70%** | **CICE** |
| --- | --- | --- | --- | --- | --- | --- | --- | --- | --- | --- | --- |
| Score | LightGBM | 0.321 | 3.863 | 24.251 | 4.909 | 0.068 | 0.313 | 0.605 | 0.225 | 0.760 | 0.608 |
|  | rrBLUP | 0.294 | 3.854 | 24.208 | 4.902 | 0.072 | 0.284 | 0.606 | 0.200 | 0.756 | 0.595 |
|  | DNNGP | 0.250 | 5.112 | 41.224 | 6.383 | -0.591 | 0.226 | 0.623 | 0.213 | 0.728 | 0.557 |
|  | RFR | 0.229 | 3.964 | 25.474 | 5.031 | 0.022 | 0.223 | 0.530 | 0.088 | 0.735 | 0.561 |
|  | SVR | 0.222 | 4.399 | 30.827 | 5.533 | -0.187 | 0.197 | 0.579 | 0.225 | 0.737 | 0.552 |
|  | BRR | 0.053 | 4.212 | 27.882 | 5.270 | -0.079 | 0.040 | 0.501 | 0.100 | 0.682 | 0.470 |
|  | BayesA | 0.038 | 4.456 | 30.775 | 5.536 | -0.194 | 0.015 | 0.486 | 0.125 | 0.670 | 0.459 |
|  | BL | 0.024 | 4.499 | 31.299 | 5.584 | -0.215 | 0.001 | 0.467 | 0.125 | 0.670 | 0.452 |
| Ranking | LightGBM | 1 | 2 | 2 | 2 | 2 | 1 | 3 | 1 | 1 | 1 |
|  | rrBLUP | 2 | 1 | 1 | 1 | 1 | 2 | 2 | 4 | 2 | 2 |
|  | DNNGP | 3 | 8 | 8 | 8 | 8 | 3 | 1 | 3 | 5 | 4 |
|  | RFR | 4 | 3 | 3 | 3 | 3 | 4 | 5 | 8 | 4 | 3 |
|  | SVR | 5 | 5 | 6 | 5 | 5 | 5 | 4 | 1 | 3 | 5 |
|  | BRR | 6 | 4 | 4 | 4 | 4 | 6 | 6 | 7 | 6 | 6 |
|  | BayesA | 7 | 6 | 5 | 6 | 6 | 7 | 7 | 5 | 7 | 7 |
|  | BL | 8 | 7 | 7 | 7 | 7 | 8 | 8 | 5 | 7 | 8 |

PGW, per plant grain weight; PCC, Pearson’s correlation coefficient; MAE, mean absolute error; MSE, mean squared error; RMSE, root mean squared error; R-squared, coefficient of determination R²; SRCC, Spearman's rank correlation coefficient; NDCG@5, top 5 normalized discounted cumulative gain; THR@10%, top 10% hit rate; BHR@70%, bottom 70% hit rate; CICE, combined index for correlation and error. rrBLUP, ridge regression best linear unbiased prediction; BL, Bayesian LASSO; BRR, Bayesian ridge regression; LightGBM, light gradient boosting machine; SVR, support vector regression; RF, random forest; DNNGP, deep neural network for genomic prediction.

**Supplementary Table S21.** Score and ranking of eight prediction methods on PCC and nine other evaluation metrics for the trait PH on the test set (20%) of the IRRI dataset.

|  | **Method** | **PCC** | **MAE** | **MSE** | **RMSE** | **R-squared** | **SRCC** | **NDCG@5** | **THR@10%** | **BHR@70%** | **CICE** |
| --- | --- | --- | --- | --- | --- | --- | --- | --- | --- | --- | --- |
| Score | rrBLUP | 0.485 | 4.448 | 33.520 | 5.790 | 0.207 | 0.509 | 0.934 | 0.167 | 0.821 | 0.699 |
|  | SVR | 0.387 | 6.011 | 80.861 | 8.992 | -0.912 | 0.559 | 0.909 | 0.167 | 0.769 | 0.635 |
|  | LightGBM | 0.349 | 4.846 | 39.508 | 6.286 | 0.066 | 0.383 | 0.934 | 0.333 | 0.795 | 0.627 |
|  | RFR | 0.286 | 5.001 | 41.902 | 6.473 | 0.009 | 0.301 | 0.934 | 0.167 | 0.795 | 0.594 |
|  | DNNGP | 0.130 | 95.740 | 9208.239 | 95.960 | -216.760 | 0.147 | 0.897 | 0.167 | 0.641 | 0.134 |
|  | BRR | 0.056 | 5.784 | 53.590 | 7.321 | -0.267 | 0.008 | 0.882 | 0.000 | 0.667 | 0.472 |
|  | BayesA | 0.005 | 5.985 | 59.194 | 7.694 | -0.400 | -0.027 | 0.877 | 0.000 | 0.692 | 0.444 |
|  | BL | -0.023 | 6.271 | 65.993 | 8.124 | -0.561 | -0.059 | 0.904 | 0.000 | 0.667 | 0.428 |
| Ranking | rrBLUP | 1 | 1 | 1 | 1 | 1 | 2 | 2 | 2 | 1 | 1 |
|  | SVR | 2 | 6 | 7 | 7 | 7 | 1 | 4 | 2 | 4 | 2 |
|  | LightGBM | 3 | 2 | 2 | 2 | 2 | 3 | 1 | 1 | 2 | 3 |
|  | RFR | 4 | 3 | 3 | 3 | 3 | 4 | 3 | 2 | 2 | 4 |
|  | DNNGP | 5 | 8 | 8 | 8 | 8 | 5 | 6 | 2 | 8 | 8 |
|  | BRR | 6 | 4 | 4 | 4 | 4 | 6 | 7 | 6 | 6 | 5 |
|  | BayesA | 7 | 5 | 5 | 5 | 5 | 7 | 8 | 6 | 5 | 6 |
|  | BL | 8 | 7 | 6 | 6 | 6 | 8 | 5 | 6 | 6 | 7 |

PH*,* plant height; PCC, Pearson’s correlation coefficient; MAE, mean absolute error; MSE, mean squared error; RMSE, root mean squared error; R-squared, coefficient of determination R²; SRCC, Spearman's rank correlation coefficient; NDCG@5, top 5 normalized discounted cumulative gain; THR@10%, top 10% hit rate; BHR@70%, bottom 70% hit rate; CICE, combined index for correlation and error. rrBLUP, ridge regression best linear unbiased prediction; BL, Bayesian LASSO; BRR, Bayesian ridge regression; LightGBM, light gradient boosting machine; SVR, support vector regression; RF, random forest; DNNGP, deep neural network for genomic prediction.

**Supplementary Table S22.** Score and ranking of eight prediction methods on PCC and nine other evaluation metrics for the trait FLW on the test set (20%) of the IRRI dataset.

|  | **Method** | **PCC** | **MAE** | **MSE** | **RMSE** | **R-squared** | **SRCC** | **NDCG@5** | **THR@10%** | **BHR@70%** | **CICE** |
| --- | --- | --- | --- | --- | --- | --- | --- | --- | --- | --- | --- |
| Score | LightGBM | 0.707 | 2.324 | 10.623 | 3.259 | 0.499 | 0.712 | 0.960 | 0.500 | 0.872 | 0.799 |
|  | RFR | 0.667 | 2.877 | 11.807 | 3.436 | 0.443 | 0.626 | 0.924 | 0.167 | 0.795 | 0.767 |
|  | rrBLUP | 0.597 | 2.879 | 13.726 | 3.705 | 0.352 | 0.586 | 0.963 | 0.167 | 0.846 | 0.731 |
|  | DNNGP | 0.416 | 75.004 | 5646.329 | 75.142 | -265.553 | 0.407 | 0.957 | 0.333 | 0.718 | 0.220 |
|  | SVR | 0.346 | 4.290 | 53.914 | 7.343 | -1.545 | 0.517 | 0.958 | 0.167 | 0.821 | 0.577 |
|  | BRR | 0.269 | 3.845 | 20.803 | 4.561 | 0.018 | 0.255 | 0.915 | 0.167 | 0.718 | 0.547 |
|  | BL | 0.265 | 4.010 | 22.933 | 4.789 | -0.083 | 0.245 | 0.929 | 0.167 | 0.718 | 0.542 |
|  | BayesA | 0.257 | 4.016 | 22.835 | 4.779 | -0.078 | 0.236 | 0.927 | 0.167 | 0.718 | 0.538 |
| Ranking | LightGBM | 1 | 1 | 1 | 1 | 1 | 1 | 2 | 1 | 1 | 1 |
|  | RFR | 2 | 2 | 2 | 2 | 2 | 2 | 7 | 3 | 4 | 2 |
|  | rrBLUP | 3 | 3 | 3 | 3 | 3 | 3 | 1 | 3 | 2 | 3 |
|  | DNNGP | 4 | 8 | 8 | 8 | 8 | 5 | 4 | 2 | 5 | 8 |
|  | SVR | 5 | 7 | 7 | 7 | 7 | 4 | 3 | 3 | 3 | 4 |
|  | BRR | 6 | 4 | 4 | 4 | 4 | 6 | 8 | 3 | 5 | 5 |
|  | BL | 7 | 5 | 6 | 6 | 6 | 7 | 5 | 3 | 5 | 6 |
|  | BayesA | 8 | 6 | 5 | 5 | 5 | 8 | 6 | 3 | 5 | 7 |

FLW, flowering date; PCC, Pearson’s correlation coefficient; MAE, mean absolute error; MSE, mean squared error; RMSE, root mean squared error; R-squared, coefficient of determination R²; SRCC, Spearman's rank correlation coefficient; NDCG@5, top 5 normalized discounted cumulative gain; THR@10%, top 10% hit rate; BHR@70%, bottom 70% hit rate; CICE, combined index for correlation and error. rrBLUP, ridge regression best linear unbiased prediction; BL, Bayesian LASSO; BRR, Bayesian ridge regression; LightGBM, light gradient boosting machine; SVR, support vector regression; RF, random forest; DNNGP, deep neural network for genomic prediction.

**Supplementary Table S23.** Score and ranking of eight prediction methods on PCC and nine other evaluation metrics for the trait GrW on the test set (20%) of the IRRI dataset.

|  | **Method** | **PCC** | **MAE** | **MSE** | **RMSE** | **R-squared** | **SRCC** | **NDCG@5** | **THR@10%** | **BHR@70%** | **CICE** |
| --- | --- | --- | --- | --- | --- | --- | --- | --- | --- | --- | --- |
| Score | rrBLUP | 0.353 | 0.069 | 0.007 | 0.082 | 0.116 | 0.236 | 0.972 | 0.167 | 0.718 | 0.630 |
|  | LightGBM | 0.288 | 0.071 | 0.007 | 0.086 | 0.033 | 0.221 | 0.972 | 0.167 | 0.692 | 0.596 |
|  | RFR | 0.207 | 0.068 | 0.007 | 0.086 | 0.039 | 0.090 | 0.966 | 0.000 | 0.667 | 0.557 |
|  | SVR | 0.103 | 0.091 | 0.014 | 0.116 | -0.763 | 0.100 | 0.961 | 0.000 | 0.718 | 0.490 |
|  | DNNGP | 0.054 | 2.204 | 4.934 | 2.221 | -643.985 | 0.061 | 0.972 | 0.167 | 0.718 | 0.049 |
|  | BayesA | -0.017 | 0.090 | 0.012 | 0.109 | -0.538 | -0.025 | 0.955 | 0.333 | 0.692 | 0.431 |
|  | BL | -0.018 | 0.084 | 0.010 | 0.100 | -0.309 | -0.035 | 0.971 | 0.333 | 0.692 | 0.434 |
|  | BRR | -0.025 | 0.083 | 0.010 | 0.099 | -0.278 | -0.022 | 0.950 | 0.333 | 0.692 | 0.432 |
| Ranking | rrBLUP | 1 | 2 | 1 | 1 | 1 | 1 | 1 | 4 | 1 | 1 |
|  | LightGBM | 2 | 3 | 2 | 3 | 3 | 2 | 1 | 4 | 4 | 2 |
|  | RFR | 3 | 1 | 2 | 2 | 2 | 4 | 5 | 7 | 8 | 3 |
|  | SVR | 4 | 7 | 7 | 7 | 7 | 3 | 6 | 7 | 1 | 4 |
|  | DNNGP | 5 | 8 | 8 | 8 | 8 | 5 | 1 | 4 | 1 | 8 |
|  | BayesA | 6 | 6 | 6 | 6 | 6 | 7 | 7 | 1 | 4 | 7 |
|  | BL | 7 | 5 | 5 | 5 | 5 | 8 | 4 | 1 | 4 | 5 |
|  | BRR | 8 | 4 | 4 | 4 | 4 | 6 | 8 | 1 | 4 | 6 |

GrW, grain width; PCC, Pearson’s correlation coefficient; MAE, mean absolute error; MSE, mean squared error; RMSE, root mean squared error; R-squared, coefficient of determination R²; SRCC, Spearman's rank correlation coefficient; NDCG@5, top 5 normalized discounted cumulative gain; THR@10%, top 10% hit rate; BHR@70%, bottom 70% hit rate; CICE, combined index for correlation and error. rrBLUP, ridge regression best linear unbiased prediction; BL, Bayesian LASSO; BRR, Bayesian ridge regression; LightGBM, light gradient boosting machine; SVR, support vector regression; RF, random forest; DNNGP, deep neural network for genomic prediction.

**Supplementary Table S24.** Score and ranking of eight prediction methods on PCC and nine other evaluation metrics for the trait PedL on the test set (20%) of the IRRI dataset.

|  | **Method** | **PCC** | **MAE** | **MSE** | **RMSE** | **R-squared** | **SRCC** | **NDCG@5** | **THR@10%** | **BHR@70%** | **CICE** |
| --- | --- | --- | --- | --- | --- | --- | --- | --- | --- | --- | --- |
| Score | rrBLUP | 0.306 | 0.792 | 1.097 | 1.047 | 0.062 | 0.301 | 0.621 | 0.000 | 0.769 | 0.577 |
|  | RFR | 0.202 | 0.829 | 1.158 | 1.076 | 0.009 | 0.125 | 0.714 | 0.167 | 0.667 | 0.522 |
|  | LightGBM | 0.176 | 0.825 | 1.185 | 1.088 | -0.014 | 0.272 | 0.548 | 0.000 | 0.744 | 0.509 |
|  | SVR | -0.023 | 1.064 | 1.948 | 1.396 | -0.667 | 0.102 | 0.499 | 0.000 | 0.667 | 0.389 |
|  | DNNGP | -0.066 | 3.159 | 11.151 | 3.339 | -8.543 | -0.055 | 0.532 | 0.000 | 0.641 | 0.226 |
|  | BRR | -0.101 | 0.917 | 1.418 | 1.191 | -0.213 | -0.110 | 0.483 | 0.000 | 0.641 | 0.363 |
|  | BayesA | -0.103 | 0.971 | 1.529 | 1.236 | -0.308 | -0.119 | 0.494 | 0.000 | 0.667 | 0.357 |
|  | BL | -0.118 | 0.959 | 1.515 | 1.231 | -0.297 | -0.133 | 0.495 | 0.000 | 0.641 | 0.351 |
| Ranking | rrBLUP | 1 | 1 | 1 | 1 | 1 | 1 | 2 | 2 | 1 | 1 |
|  | RFR | 2 | 3 | 2 | 2 | 2 | 3 | 1 | 1 | 3 | 2 |
|  | LightGBM | 3 | 2 | 3 | 3 | 3 | 2 | 3 | 2 | 2 | 3 |
|  | SVR | 4 | 7 | 7 | 7 | 7 | 4 | 5 | 2 | 3 | 4 |
|  | DNNGP | 5 | 8 | 8 | 8 | 8 | 5 | 4 | 2 | 6 | 8 |
|  | BRR | 6 | 4 | 4 | 4 | 4 | 6 | 8 | 2 | 6 | 5 |
|  | BayesA | 7 | 6 | 6 | 6 | 6 | 7 | 7 | 2 | 3 | 6 |
|  | BL | 8 | 5 | 5 | 5 | 5 | 8 | 6 | 2 | 6 | 7 |

PedL, peduncle length; PCC, Pearson’s correlation coefficient; MAE, mean absolute error; MSE, mean squared error; RMSE, root mean squared error; R-squared, coefficient of determination R²; SRCC, Spearman's rank correlation coefficient; NDCG@5, top 5 normalized discounted cumulative gain; THR@10%, top 10% hit rate; BHR@70%, bottom 70% hit rate; CICE, combined index for correlation and error. rrBLUP, ridge regression best linear unbiased prediction; BL, Bayesian LASSO; BRR, Bayesian ridge regression; LightGBM, light gradient boosting machine; SVR, support vector regression; RF, random forest; DNNGP, deep neural network for genomic prediction.

**Supplementary Table S25.** Score and ranking of eight prediction methods on PCC and nine other evaluation metrics for the trait YLD on the test set (20%) of the IRRI dataset.

|  | **Method** | **PCC** | **MAE** | **MSE** | **RMSE** | **R-squared** | **SRCC** | **NDCG@5** | **THR@10%** | **BHR@70%** | **CICE** |
| --- | --- | --- | --- | --- | --- | --- | --- | --- | --- | --- | --- |
| Score | BRR | 0.906 | 419.634 | 296166.700 | 544.212 | 0.229 | 0.886 | 0.990 | 0.667 | 0.897 | 0.907 |
|  | BayesA | 0.711 | 442.182 | 329594.900 | 574.104 | 0.142 | 0.678 | 0.917 | 0.500 | 0.846 | 0.807 |
|  | DNNGP | 0.249 | 4010.322 | 16463424.950 | 4057.515 | -41.866 | 0.257 | 0.832 | 0.333 | 0.718 | 0.324 |
|  | rrBLUP | 0.238 | 479.945 | 369696.100 | 608.026 | 0.037 | 0.154 | 0.897 | 0.333 | 0.718 | 0.567 |
|  | BL | 0.201 | 613.433 | 541839.200 | 736.097 | -0.411 | 0.200 | 0.837 | 0.167 | 0.769 | 0.535 |
|  | LightGBM | 0.115 | 550.755 | 476822.800 | 690.524 | -0.242 | 0.035 | 0.874 | 0.167 | 0.667 | 0.498 |
|  | RFR | 0.046 | 471.948 | 392344.300 | 626.374 | -0.022 | 0.027 | 0.818 | 0.167 | 0.718 | 0.472 |
|  | SVR | -0.016 | 967.649 | 2335678.000 | 1528.292 | -5.082 | 0.036 | 0.764 | 0.000 | 0.641 | 0.392 |
| Ranking | BRR | 1 | 1 | 1 | 1 | 1 | 1 | 1 | 1 | 1 | 1 |
|  | BayesA | 2 | 2 | 2 | 2 | 2 | 2 | 2 | 2 | 2 | 2 |
|  | DNNGP | 3 | 8 | 8 | 8 | 8 | 3 | 6 | 3 | 4 | 8 |
|  | rrBLUP | 4 | 4 | 3 | 3 | 3 | 5 | 3 | 3 | 4 | 3 |
|  | BL | 5 | 6 | 6 | 6 | 6 | 4 | 5 | 5 | 3 | 4 |
|  | LightGBM | 6 | 5 | 5 | 5 | 5 | 7 | 4 | 5 | 7 | 5 |
|  | RFR | 7 | 3 | 4 | 4 | 4 | 8 | 7 | 5 | 4 | 6 |
|  | SVR | 8 | 7 | 7 | 7 | 7 | 6 | 8 | 8 | 8 | 7 |

YLD, grain yield; PCC, Pearson’s correlation coefficient; MAE, mean absolute error; MSE, mean squared error; RMSE, root mean squared error; R-squared, coefficient of determination R²; SRCC, Spearman's rank correlation coefficient; NDCG@5, top 5 normalized discounted cumulative gain; THR@10%, top 10% hit rate; BHR@70%, bottom 70% hit rate; CICE, combined index for correlation and error. rrBLUP, ridge regression best linear unbiased prediction; BL, Bayesian LASSO; BRR, Bayesian ridge regression; LightGBM, light gradient boosting machine; SVR, support vector regression; RF, random forest; DNNGP, deep neural network for genomic prediction.

**Supplementary Table S26.** Score and ranking of eight prediction methods on PCC and nine other evaluation metrics for the trait Exs on the test set (20%) of the IRRI dataset.

|  | **Method** | **PCC** | **MAE** | **MSE** | **RMSE** | **R-squared** | **SRCC** | **NDCG@5** | **THR@10%** | **BHR@70%** | **CICE** |
| --- | --- | --- | --- | --- | --- | --- | --- | --- | --- | --- | --- |
| Score | DNNGP | 0.198 | 5.201 | 27.298 | 5.225 | -7334.584 | 0.156 | 0.903 | 0.333 | 0.641 | 0.099 |
|  | LightGBM | 0.169 | 0.035 | 0.004 | 0.062 | -0.037 | 0.122 | 0.885 | 0.167 | 0.744 | 0.529 |
|  | rrBLUP | 0.164 | 0.033 | 0.004 | 0.061 | -0.001 | 0.065 | 0.906 | 0.167 | 0.744 | 0.530 |
|  | RFR | 0.004 | 0.035 | 0.004 | 0.063 | -0.055 | -0.001 | 0.851 | 0.000 | 0.769 | 0.447 |
|  | SVR | -0.017 | 0.057 | 0.008 | 0.088 | -1.084 | 0.016 | 0.860 | 0.000 | 0.667 | 0.406 |
|  | BayesA | -0.053 | 0.034 | 0.004 | 0.065 | -0.148 | 0.062 | 0.851 | 0.167 | 0.744 | 0.420 |
|  | BRR | -0.059 | 0.032 | 0.004 | 0.064 | -0.093 | 0.063 | 0.851 | 0.167 | 0.744 | 0.420 |
|  | BL | -0.083 | 0.033 | 0.004 | 0.064 | -0.110 | -0.001 | 0.851 | 0.167 | 0.744 | 0.406 |
| Ranking | DNNGP | 1 | 8 | 8 | 8 | 8 | 1 | 2 | 1 | 8 | 8 |
|  | LightGBM | 2 | 5 | 2 | 2 | 2 | 2 | 3 | 2 | 2 | 2 |
|  | rrBLUP | 3 | 2 | 1 | 1 | 1 | 3 | 1 | 2 | 2 | 1 |
|  | RFR | 4 | 5 | 2 | 3 | 3 | 8 | 5 | 7 | 1 | 3 |
|  | SVR | 5 | 7 | 7 | 7 | 7 | 6 | 4 | 7 | 7 | 7 |
|  | BayesA | 6 | 4 | 6 | 6 | 6 | 5 | 5 | 2 | 2 | 4 |
|  | BRR | 7 | 1 | 4 | 4 | 4 | 4 | 5 | 2 | 2 | 5 |
|  | BL | 8 | 3 | 4 | 5 | 5 | 7 | 5 | 2 | 2 | 6 |

Exs, panicle exertion rate; PCC, Pearson’s correlation coefficient; MAE, mean absolute error; MSE, mean squared error; RMSE, root mean squared error; R-squared, coefficient of determination R²; SRCC, Spearman's rank correlation coefficient; NDCG@5, top 5 normalized discounted cumulative gain; THR@10%, top 10% hit rate; BHR@70%, bottom 70% hit rate; CICE, combined index for correlation and error. rrBLUP, ridge regression best linear unbiased prediction; BL, Bayesian LASSO; BRR, Bayesian ridge regression; LightGBM, light gradient boosting machine; SVR, support vector regression; RF, random forest; DNNGP, deep neural network for genomic prediction.

**Supplementary Table S27.** Score and ranking of eight prediction methods on PCC and nine other evaluation metrics for the trait Lg on the test set (20%) of the IRRI dataset.

|  | **Method** | **PCC** | **MAE** | **MSE** | **RMSE** | **R-squared** | **SRCC** | **NDCG@5** | **THR@10%** | **BHR@70%** | **CICE** |
| --- | --- | --- | --- | --- | --- | --- | --- | --- | --- | --- | --- |
| Score | DNNGP | 0.018 | 0.424 | 0.571 | 0.756 | -0.258 | -0.027 | 0.372 | 0.333 | 0.718 | 0.461 |
|  | BRR | -0.016 | 0.369 | 0.507 | 0.712 | -0.117 | 0.068 | 0.342 | 0.000 | 0.718 | 0.450 |
|  | rrBLUP | -0.040 | 0.380 | 0.473 | 0.688 | -0.043 | -0.078 | 0.313 | 0.167 | 0.769 | 0.437 |
|  | BL | -0.044 | 0.382 | 0.535 | 0.732 | -0.179 | 0.017 | 0.342 | 0.000 | 0.692 | 0.435 |
|  | BayesA | -0.045 | 0.394 | 0.558 | 0.747 | -0.228 | 0.022 | 0.338 | 0.000 | 0.718 | 0.433 |
|  | SVR | -0.057 | 0.637 | 0.920 | 0.959 | -1.027 | -0.095 | 0.313 | 0.000 | 0.718 | 0.401 |
|  | LightGBM | -0.058 | 0.409 | 0.543 | 0.737 | -0.197 | -0.181 | 0.313 | 0.000 | 0.744 | 0.425 |
|  | RFR | -0.131 | 0.383 | 0.510 | 0.714 | -0.123 | -0.124 | 0.313 | 0.000 | 0.769 | 0.391 |
| Ranking | DNNGP | 1 | 7 | 7 | 7 | 7 | 4 | 1 | 1 | 4 | 1 |
|  | BRR | 2 | 1 | 2 | 2 | 2 | 1 | 2 | 3 | 4 | 2 |
|  | rrBLUP | 3 | 2 | 1 | 1 | 1 | 5 | 5 | 2 | 1 | 3 |
|  | BL | 4 | 3 | 4 | 4 | 4 | 3 | 2 | 3 | 8 | 4 |
|  | BayesA | 5 | 5 | 6 | 6 | 6 | 2 | 4 | 3 | 4 | 5 |
|  | SVR | 6 | 8 | 8 | 8 | 8 | 6 | 5 | 3 | 4 | 7 |
|  | LightGBM | 7 | 6 | 5 | 5 | 5 | 8 | 5 | 3 | 3 | 6 |
|  | RFR | 8 | 4 | 3 | 3 | 3 | 7 | 5 | 3 | 1 | 8 |

Lg, lodging score; PCC, Pearson’s correlation coefficient; MAE, mean absolute error; MSE, mean squared error; RMSE, root mean squared error; R-squared, coefficient of determination R²; SRCC, Spearman's rank correlation coefficient; NDCG@5, top 5 normalized discounted cumulative gain; THR@10%, top 10% hit rate; BHR@70%, bottom 70% hit rate; CICE, combined index for correlation and error. rrBLUP, ridge regression best linear unbiased prediction; BL, Bayesian LASSO; BRR, Bayesian ridge regression; LightGBM, light gradient boosting machine; SVR, support vector regression; RF, random forest; DNNGP, deep neural network for genomic prediction.

**Supplementary Table S28.** Score and ranking of eight prediction methods on PCC and nine other evaluation metrics for the trait GY on the test set (20%) of the wheat599 dataset with 1279 markers (without dimensionality reduction).

|  | **Method** | **PCC** | **MAE** | **MSE** | **RMSE** | **R-squared** | **SRCC** | **NDCG@5** | **THR@10%** | **BHR@70%** | **CICE** |
| --- | --- | --- | --- | --- | --- | --- | --- | --- | --- | --- | --- |
| Score | BayesA | 0.726 | 0.577 | 0.539 | 0.734 | 0.424 | 0.674 | 0.743 | 0.500 | 0.833 | 0.815 |
|  | BL | 0.672 | 0.5990 | 0.582 | 0.763 | 0.377 | 0.614 | 0.672 | 0.417 | 0.810 | 0.786 |
|  | DNNGP | 0.649 | 0.967 | 1.493 | 1.222 | -0.597 | 0.658 | 0.584 | 0.250 | 0.845 | 0.747 |
|  | RFR | 0.609 | 0.600 | 0.600 | 0.775 | 0.358 | 0.596 | 0.515 | 0.333 | 0.810 | 0.754 |
|  | rrBLUP | 0.596 | 0.5988 | 0.609 | 0.781 | 0.348 | 0.606 | 0.504 | 0.167 | 0.845 | 0.748 |
|  | LightGBM | 0.560 | 0.620 | 0.667 | 0.817 | 0.286 | 0.554 | 0.520 | 0.250 | 0.774 | 0.728 |
|  | SVR | 0.542 | 0.643 | 0.723 | 0.850 | 0.227 | 0.555 | 0.431 | 0.167 | 0.821 | 0.717 |
|  | BRR | 0.499 | 0.658 | 0.713 | 0.844 | 0.237 | 0.444 | 0.295 | 0.250 | 0.762 | 0.695 |
| Ranking | BayesA | 1 | 1 | 1 | 1 | 1 | 1 | 1 | 1 | 3 | 1 |
|  | BL | 2 | 3 | 2 | 2 | 2 | 3 | 2 | 2 | 5 | 2 |
|  | DNNGP | 3 | 8 | 8 | 8 | 8 | 2 | 3 | 4 | 1 | 5 |
|  | RFR | 4 | 4 | 3 | 3 | 3 | 5 | 5 | 3 | 5 | 3 |
|  | rrBLUP | 5 | 2 | 4 | 4 | 4 | 4 | 6 | 7 | 1 | 4 |
|  | LightGBM | 6 | 5 | 5 | 5 | 5 | 7 | 4 | 4 | 7 | 6 |
|  | SVR | 7 | 6 | 7 | 7 | 7 | 6 | 7 | 7 | 4 | 7 |
|  | BRR | 8 | 7 | 6 | 6 | 6 | 8 | 8 | 4 | 8 | 8 |

GY*,* average grain yield; PCC, Pearson’s correlation coefficient; MAE, mean absolute error; MSE, mean squared error; RMSE, root mean squared error; R-squared, coefficient of determination R²; SRCC, Spearman's rank correlation coefficient; NDCG@5, top 5 normalized discounted cumulative gain; THR@10%, top 10% hit rate; BHR@70%, bottom 70% hit rate; CICE, combined index for correlation and error. rrBLUP, ridge regression best linear unbiased prediction; BL, Bayesian LASSO; BRR, Bayesian ridge regression; LightGBM, light gradient boosting machine; SVR, support vector regression; RF, random forest; DNNGP, deep neural network for genomic prediction.

**Supplementary Table S29.** Score and ranking of eight prediction methods on PCC and nine other evaluation metrics for the trait GY on the test set (20%) of the wheat599 dataset with 251 principal components after dimensionality reduction.

|  | **Method** | **PCC** | **MAE** | **MSE** | **RMSE** | **R-squared** | **SRCC** | **NDCG@5** | **THR@10%** | **BHR@70%** | **CICE** |
| --- | --- | --- | --- | --- | --- | --- | --- | --- | --- | --- | --- |
| Score | BayesA | 0.731 | 0.575 | 0.535 | 0.731 | 0.428 | 0.678 | 0.768 | 0.500 | 0.821 | 0.818 |
|  | BL | 0.684 | 0.602 | 0.581 | 0.762 | 0.379 | 0.628 | 0.622 | 0.417 | 0.798 | 0.792 |
|  | DNNGP | 0.610 | 1.178 | 2.022 | 1.422 | -1.163 | 0.595 | 0.235 | 0.167 | 0.786 | 0.712 |
|  | rrBLUP | 0.591 | 0.603 | 0.616 | 0.785 | 0.341 | 0.601 | 0.452 | 0.167 | 0.833 | 0.745 |
|  | RFR | 0.525 | 0.627 | 0.700 | 0.837 | 0.251 | 0.553 | 0.243 | 0.250 | 0.833 | 0.710 |
|  | LightGBM | 0.523 | 0.623 | 0.683 | 0.826 | 0.269 | 0.533 | 0.258 | 0.250 | 0.845 | 0.710 |
|  | BRR | 0.504 | 0.662 | 0.711 | 0.843 | 0.240 | 0.440 | 0.318 | 0.250 | 0.762 | 0.697 |
|  | SVR | 0.458 | 0.656 | 0.740 | 0.860 | 0.208 | 0.479 | 0.343 | 0.083 | 0.810 | 0.675 |
| Ranking | BayesA | 1 | 1 | 1 | 1 | 1 | 1 | 1 | 1 | 4 | 1 |
|  | BL | 2 | 2 | 2 | 2 | 2 | 2 | 2 | 2 | 6 | 2 |
|  | DNNGP | 3 | 8 | 8 | 8 | 8 | 4 | 8 | 6 | 7 | 4 |
|  | rrBLUP | 4 | 3 | 3 | 3 | 3 | 3 | 3 | 6 | 2 | 3 |
|  | RFR | 5 | 5 | 5 | 5 | 5 | 5 | 7 | 3 | 2 | 5 |
|  | LightGBM | 6 | 4 | 4 | 4 | 4 | 6 | 6 | 3 | 1 | 6 |
|  | BRR | 7 | 7 | 6 | 6 | 6 | 8 | 5 | 3 | 8 | 7 |
|  | SVR | 8 | 6 | 7 | 7 | 7 | 7 | 4 | 8 | 5 | 8 |

GY*,* average grain yield; PCC, Pearson’s correlation coefficient; MAE, mean absolute error; MSE, mean squared error; RMSE, root mean squared error; R-squared, coefficient of determination R²; SRCC, Spearman's rank correlation coefficient; NDCG@5, top 5 normalized discounted cumulative gain; THR@10%, top 10% hit rate; BHR@70%, bottom 70% hit rate; CICE, combined index for correlation and error. rrBLUP, ridge regression best linear unbiased prediction; BL, Bayesian LASSO; BRR, Bayesian ridge regression; LightGBM, light gradient boosting machine; SVR, support vector regression; RF, random forest; DNNGP, deep neural network for genomic prediction.

**Supplementary Table S30.** Score and ranking of eight prediction methods on PCC and nine other evaluation metrics for the trait Biomass on the test set (20%) of the wheat487 dataset.

|  | **Method** | **PCC** | **MAE** | **MSE** | **RMSE** | **R-squared** | **SRCC** | **NDCG@5** | **THR@10%** | **BHR@70%** | **CICE** |
| --- | --- | --- | --- | --- | --- | --- | --- | --- | --- | --- | --- |
| Score | DNNGP | 0.665 | 0.308 | 0.108 | 0.328 | -42.137 | 0.619 | 0.766 | 0.100 | 0.809 | 0.473 |
|  | rrBLUP | 0.654 | 0.029 | 0.001 | 0.038 | 0.424 | 0.629 | 0.797 | 0.200 | 0.824 | 0.771 |
|  | LightGBM | 0.619 | 0.031 | 0.002 | 0.040 | 0.366 | 0.590 | 0.900 | 0.300 | 0.824 | 0.750 |
|  | RFR | 0.614 | 0.033 | 0.002 | 0.040 | 0.345 | 0.612 | 0.800 | 0.100 | 0.824 | 0.743 |
|  | SVR | 0.580 | 0.032 | 0.002 | 0.043 | 0.273 | 0.570 | 0.792 | 0.100 | 0.809 | 0.729 |
|  | BRR | 0.009 | 0.044 | 0.003 | 0.052 | -0.089 | 0.043 | 0.574 | 0.200 | 0.721 | 0.422 |
|  | BL | 0.002 | 0.044 | 0.003 | 0.052 | -0.090 | 0.046 | 0.621 | 0.200 | 0.735 | 0.418 |
|  | BayesA | -0.042 | 0.045 | 0.003 | 0.055 | -0.217 | -0.006 | 0.612 | 0.200 | 0.706 | 0.394 |
| Ranking | DNNGP | 1 | 8 | 8 | 8 | 8 | 2 | 5 | 6 | 4 | 5 |
|  | rrBLUP | 2 | 1 | 1 | 1 | 1 | 1 | 3 | 2 | 1 | 1 |
|  | LightGBM | 3 | 2 | 2 | 2 | 2 | 4 | 1 | 1 | 1 | 2 |
|  | RFR | 4 | 4 | 2 | 3 | 3 | 3 | 2 | 6 | 1 | 3 |
|  | SVR | 5 | 3 | 4 | 4 | 4 | 5 | 4 | 6 | 4 | 4 |
|  | BRR | 6 | 5 | 5 | 5 | 5 | 7 | 8 | 2 | 7 | 6 |
|  | BL | 7 | 6 | 5 | 5 | 6 | 6 | 6 | 2 | 6 | 7 |
|  | BayesA | 8 | 7 | 7 | 7 | 7 | 8 | 7 | 2 | 8 | 8 |

PCC, Pearson’s correlation coefficient; MAE, mean absolute error; MSE, mean squared error; RMSE, root mean squared error; R-squared, coefficient of determination R²; SRCC, Spearman's rank correlation coefficient; NDCG@5, top 5 normalized discounted cumulative gain; THR@10%, top 10% hit rate; BHR@70%, bottom 70% hit rate; CICE, combined index for correlation and error. rrBLUP, ridge regression best linear unbiased prediction; BL, Bayesian LASSO; BRR, Bayesian ridge regression; LightGBM, light gradient boosting machine; SVR, support vector regression; RF, random forest; DNNGP, deep neural network for genomic prediction.

**Supplementary Table S31.** Score and ranking of eight prediction methods on PCC and nine other evaluation metrics for the trait Yield on the test set (20%) of the wheat487 dataset.

|  | **Method** | **PCC** | **MAE** | **MSE** | **RMSE** | **R-squared** | **SRCC** | **NDCG@5** | **THR@10%** | **BHR@70%** | **CICE** |
| --- | --- | --- | --- | --- | --- | --- | --- | --- | --- | --- | --- |
| Score | LightGBM | 0.743 | 0.351 | 0.240 | 0.490 | 0.542 | 0.721 | 0.924 | 0.300 | 0.824 | 0.813 |
|  | DNNGP | 0.742 | 0.429 | 0.347 | 0.589 | 0.340 | 0.695 | 0.910 | 0.300 | 0.824 | 0.801 |
|  | rrBLUP | 0.726 | 0.360 | 0.250 | 0.500 | 0.524 | 0.683 | 0.805 | 0.300 | 0.868 | 0.803 |
|  | SVR | 0.700 | 0.367 | 0.272 | 0.521 | 0.483 | 0.676 | 0.777 | 0.300 | 0.838 | 0.789 |
|  | RFR | 0.637 | 0.455 | 0.325 | 0.570 | 0.381 | 0.653 | 0.798 | 0.100 | 0.794 | 0.745 |
|  | BRR | 0.024 | 0.641 | 0.562 | 0.749 | -0.070 | 0.021 | 0.604 | 0.200 | 0.691 | 0.411 |
|  | BL | -0.007 | 0.650 | 0.580 | 0.762 | -0.105 | -0.024 | 0.584 | 0.300 | 0.691 | 0.394 |
|  | BayesA | -0.051 | 0.672 | 0.639 | 0.799 | -0.217 | -0.062 | 0.606 | 0.300 | 0.662 | 0.370 |
| Ranking | LightGBM | 1 | 1 | 1 | 1 | 1 | 1 | 1 | 1 | 3 | 1 |
|  | DNNGP | 2 | 4 | 5 | 5 | 5 | 2 | 2 | 1 | 3 | 3 |
|  | rrBLUP | 3 | 2 | 2 | 2 | 2 | 3 | 3 | 1 | 1 | 2 |
|  | SVR | 4 | 3 | 3 | 3 | 3 | 4 | 5 | 1 | 2 | 4 |
|  | RFR | 5 | 5 | 4 | 4 | 4 | 5 | 4 | 8 | 5 | 5 |
|  | BRR | 6 | 6 | 6 | 6 | 6 | 6 | 7 | 7 | 6 | 6 |
|  | BL | 7 | 7 | 7 | 7 | 7 | 7 | 8 | 1 | 6 | 7 |
|  | BayesA | 8 | 8 | 8 | 8 | 8 | 8 | 6 | 1 | 8 | 8 |

PCC, Pearson’s correlation coefficient; MAE, mean absolute error; MSE, mean squared error; RMSE, root mean squared error; R-squared, coefficient of determination R²; SRCC, Spearman's rank correlation coefficient; NDCG@5, top 5 normalized discounted cumulative gain; THR@10%, top 10% hit rate; BHR@70%, bottom 70% hit rate; CICE, combined index for correlation and error. rrBLUP, ridge regression best linear unbiased prediction; BL, Bayesian LASSO; BRR, Bayesian ridge regression; LightGBM, light gradient boosting machine; SVR, support vector regression; RF, random forest; DNNGP, deep neural network for genomic prediction.

**Supplementary Table S32.** Score and ranking of eight prediction methods on PCC and nine other evaluation metrics for the trait PH on the test set (20%) of the G2F_2017 dataset.

|  | **Method** | **PCC** | **MAE** | **MSE** | **RMSE** | **R-squared** | **SRCC** | **NDCG@5** | **THR@10%** | **BHR@70%** | **CICE** |
| --- | --- | --- | --- | --- | --- | --- | --- | --- | --- | --- | --- |
| Score | rrBLUP | 0.910 | 4.653 | 35.819 | 5.985 | 0.813 | 0.901 | 0.985 | 0.571 | 0.880 | 0.929 |
|  | DNNGP | 0.897 | 33.288 | 1729.851 | 41.592 | -8.029 | 0.882 | 0.984 | 0.571 | 0.860 | 0.788 |
|  | LightGBM | 0.866 | 5.656 | 49.059 | 7.004 | 0.744 | 0.813 | 0.995 | 0.571 | 0.860 | 0.901 |
|  | SVR | 0.865 | 5.550 | 55.954 | 7.480 | 0.708 | 0.869 | 0.991 | 0.571 | 0.880 | 0.901 |
|  | RFR | 0.681 | 8.843 | 122.669 | 11.076 | 0.360 | 0.644 | 0.942 | 0.571 | 0.860 | 0.792 |
|  | BL | 0.221 | 11.800 | 198.256 | 14.080 | -0.035 | 0.248 | 0.875 | 0.286 | 0.800 | 0.546 |
|  | BayesA | 0.147 | 11.681 | 199.432 | 14.122 | -0.041 | 0.197 | 0.866 | 0.143 | 0.760 | 0.510 |
|  | BRR | 0.118 | 11.330 | 195.707 | 13.990 | -0.022 | 0.148 | 0.866 | 0.143 | 0.760 | 0.497 |
| Ranking | rrBLUP | 1 | 1 | 1 | 1 | 1 | 1 | 3 | 1 | 1 | 1 |
|  | DNNGP | 2 | 8 | 8 | 8 | 8 | 2 | 4 | 1 | 3 | 5 |
|  | LightGBM | 3 | 3 | 2 | 2 | 2 | 4 | 1 | 1 | 3 | 3 |
|  | SVR | 4 | 2 | 3 | 3 | 3 | 3 | 2 | 1 | 1 | 2 |
|  | RFR | 5 | 4 | 4 | 4 | 4 | 5 | 5 | 1 | 3 | 4 |
|  | BL | 6 | 7 | 6 | 6 | 6 | 6 | 6 | 6 | 6 | 6 |
|  | BayesA | 7 | 6 | 7 | 7 | 7 | 7 | 8 | 7 | 7 | 7 |
|  | BRR | 8 | 5 | 5 | 5 | 5 | 8 | 7 | 7 | 7 | 8 |

PH, plant height; PCC, Pearson’s correlation coefficient; MAE, mean absolute error; MSE, mean squared error; RMSE, root mean squared error; R-squared, coefficient of determination R²; SRCC, Spearman's rank correlation coefficient; NDCG@5, top 5 normalized discounted cumulative gain; THR@10%, top 10% hit rate; BHR@70%, bottom 70% hit rate; CICE, combined index for correlation and error. rrBLUP, ridge regression best linear unbiased prediction; BL, Bayesian LASSO; BRR, Bayesian ridge regression; LightGBM, light gradient boosting machine; SVR, support vector regression; RF, random forest; DNNGP, deep neural network for genomic prediction.

**Supplementary Table S33.** Score and ranking of eight prediction methods on PCC and nine other evaluation metrics for the trait Yield on the test set (20%) of the G2F_2017 dataset.

|  | **Method** | **PCC** | **MAE** | **MSE** | **RMSE** | **R-squared** | **SRCC** | **NDCG@5** | **THR@10%** | **BHR@70%** | **CICE** |
| --- | --- | --- | --- | --- | --- | --- | --- | --- | --- | --- | --- |
| Score | DNNGP | 0.627 | 13.554 | 280.298 | 16.742 | -0.753 | 0.643 | 0.937 | 0.286 | 0.780 | 0.750 |
|  | SVR | 0.594 | 7.756 | 104.074 | 10.202 | 0.349 | 0.587 | 0.972 | 0.571 | 0.780 | 0.760 |
|  | rrBLUP | 0.575 | 8.150 | 114.371 | 10.695 | 0.285 | 0.561 | 0.956 | 0.429 | 0.800 | 0.749 |
|  | LightGBM | 0.550 | 8.380 | 123.400 | 11.109 | 0.228 | 0.546 | 0.971 | 0.714 | 0.780 | 0.735 |
|  | RFR | 0.457 | 9.086 | 139.916 | 11.829 | 0.125 | 0.472 | 0.901 | 0.429 | 0.760 | 0.685 |
|  | BRR | 0.177 | 10.097 | 165.068 | 12.848 | -0.032 | 0.224 | 0.911 | 0.143 | 0.680 | 0.541 |
|  | BL | 0.101 | 10.662 | 190.026 | 13.785 | -0.188 | 0.116 | 0.876 | 0.143 | 0.660 | 0.500 |
|  | BayesA | 0.098 | 10.517 | 180.620 | 13.440 | -0.130 | 0.122 | 0.867 | 0.143 | 0.680 | 0.500 |
| Ranking | DNNGP | 1 | 8 | 8 | 8 | 8 | 1 | 4 | 5 | 2 | 2 |
|  | SVR | 2 | 1 | 1 | 1 | 1 | 2 | 1 | 2 | 2 | 1 |
|  | rrBLUP | 3 | 2 | 2 | 2 | 2 | 3 | 3 | 3 | 1 | 3 |
|  | LightGBM | 4 | 3 | 3 | 3 | 3 | 4 | 2 | 1 | 2 | 4 |
|  | RFR | 5 | 4 | 4 | 4 | 4 | 5 | 6 | 3 | 5 | 5 |
|  | BRR | 6 | 5 | 5 | 5 | 5 | 6 | 5 | 6 | 6 | 6 |
|  | BL | 7 | 7 | 7 | 7 | 7 | 8 | 7 | 6 | 8 | 7 |
|  | BayesA | 8 | 6 | 6 | 6 | 6 | 7 | 8 | 6 | 6 | 8 |

PCC, Pearson’s correlation coefficient; MAE, mean absolute error; MSE, mean squared error; RMSE, root mean squared error; R-squared, coefficient of determination R²; SRCC, Spearman's rank correlation coefficient; NDCG@5, top 5 normalized discounted cumulative gain; THR@10%, top 10% hit rate; BHR@70%, bottom 70% hit rate; CICE, combined index for correlation and error. rrBLUP, ridge regression best linear unbiased prediction; BL, Bayesian LASSO; BRR, Bayesian ridge regression; LightGBM, light gradient boosting machine; SVR, support vector regression; RF, random forest; DNNGP, deep neural network for genomic prediction.

**Supplementary Table S34.** Score and ranking of eight prediction methods on PCC and nine other evaluation metrics for the trait DTP on the test set (20%) of the CNGWAS dataset.

|  | **Method** | **PCC** | **MAE** | **MSE** | **RMSE** | **R-squared** | **SRCC** | **NDCG@5** | **THR@10%** | **BHR@70%** | **CICE** |
| --- | --- | --- | --- | --- | --- | --- | --- | --- | --- | --- | --- |
| Score | SVR | 0.617 | 2.337 | 9.106 | 3.018 | 0.379 | 0.589 | 0.935 | 0.500 | 0.817 | 0.763 |
|  | rrBLUP | 0.615 | 2.317 | 9.154 | 3.026 | 0.376 | 0.581 | 0.947 | 0.600 | 0.831 | 0.762 |
|  | DNNGP | 0.610 | 13.146 | 245.990 | 15.684 | -15.772 | 0.567 | 0.948 | 0.600 | 0.845 | 0.598 |
|  | LightGBM | 0.562 | 2.412 | 10.063 | 3.172 | 0.314 | 0.539 | 0.927 | 0.600 | 0.817 | 0.734 |
|  | RFR | 0.508 | 2.656 | 12.512 | 3.537 | 0.147 | 0.512 | 0.962 | 0.500 | 0.817 | 0.703 |
|  | BayesA | 0.340 | 2.818 | 13.296 | 3.646 | 0.093 | 0.324 | 0.925 | 0.100 | 0.775 | 0.616 |
|  | BRR | 0.339 | 2.769 | 13.060 | 3.614 | 0.110 | 0.333 | 0.908 | 0.100 | 0.761 | 0.616 |
|  | BL | 0.303 | 2.862 | 13.784 | 3.713 | 0.060 | 0.287 | 0.915 | 0.100 | 0.747 | 0.597 |
| Ranking | SVR | 1 | 2 | 1 | 1 | 1 | 1 | 4 | 4 | 3 | 1 |
|  | rrBLUP | 2 | 1 | 2 | 2 | 2 | 2 | 3 | 1 | 2 | 2 |
|  | DNNGP | 3 | 8 | 8 | 8 | 8 | 3 | 2 | 1 | 1 | 7 |
|  | LightGBM | 4 | 3 | 3 | 3 | 3 | 4 | 5 | 1 | 3 | 3 |
|  | RFR | 5 | 4 | 4 | 4 | 4 | 5 | 1 | 4 | 3 | 4 |
|  | BayesA | 6 | 6 | 6 | 6 | 6 | 7 | 6 | 6 | 6 | 6 |
|  | BRR | 7 | 5 | 5 | 5 | 5 | 6 | 8 | 6 | 7 | 5 |
|  | BL | 8 | 7 | 7 | 7 | 7 | 8 | 7 | 6 | 8 | 8 |

DTP, days to pollen; PCC, Pearson’s correlation coefficient; MAE, mean absolute error; MSE, mean squared error; RMSE, root mean squared error; R-squared, coefficient of determination R²; SRCC, Spearman's rank correlation coefficient; NDCG@5, top 5 normalized discounted cumulative gain; THR@10%, top 10% hit rate; BHR@70%, bottom 70% hit rate; CICE, combined index for correlation and error. rrBLUP, ridge regression best linear unbiased prediction; BL, Bayesian LASSO; BRR, Bayesian ridge regression; LightGBM, light gradient boosting machine; SVR, support vector regression; RF, random forest; DNNGP, deep neural network for genomic prediction.

**Supplementary Table S35.** Score and ranking of eight prediction methods on PCC and nine other evaluation metrics for the trait PH on the test set (20%) of the CNGWAS dataset.

|  | **Method** | **PCC** | **MAE** | **MSE** | **RMSE** | **R-squared** | **SRCC** | **NDCG@5** | **THR@10%** | **BHR@70%** | **CICE** |
| --- | --- | --- | --- | --- | --- | --- | --- | --- | --- | --- | --- |
| Score | SVR | 0.563 | 13.961 | 322.694 | 17.964 | 0.316 | 0.607 | 0.896 | 0.400 | 0.859 | 0.724 |
|  | rrBLUP | 0.562 | 13.795 | 324.090 | 18.003 | 0.313 | 0.598 | 0.894 | 0.400 | 0.873 | 0.723 |
|  | DNNGP | 0.541 | 22.003 | 786.601 | 28.046 | -0.668 | 0.564 | 0.893 | 0.300 | 0.817 | 0.682 |
|  | LightGBM | 0.517 | 14.079 | 349.479 | 18.694 | 0.259 | 0.565 | 0.859 | 0.300 | 0.859 | 0.700 |
|  | RFR | 0.401 | 16.520 | 438.609 | 20.943 | 0.070 | 0.434 | 0.941 | 0.400 | 0.803 | 0.633 |
|  | BayesA | 0.346 | 16.691 | 435.250 | 20.863 | 0.077 | 0.322 | 0.829 | 0.200 | 0.747 | 0.605 |
|  | BRR | 0.298 | 16.522 | 440.063 | 20.978 | 0.067 | 0.266 | 0.862 | 0.200 | 0.747 | 0.581 |
|  | BL | 0.250 | 18.109 | 505.918 | 22.493 | -0.073 | 0.213 | 0.843 | 0.100 | 0.732 | 0.551 |
| Ranking | SVR | 1 | 2 | 1 | 1 | 1 | 1 | 2 | 1 | 2 | 1 |
|  | rrBLUP | 2 | 1 | 2 | 2 | 2 | 2 | 3 | 1 | 1 | 2 |
|  | DNNGP | 3 | 8 | 8 | 8 | 8 | 4 | 4 | 4 | 4 | 4 |
|  | LightGBM | 4 | 3 | 3 | 3 | 3 | 3 | 6 | 4 | 2 | 3 |
|  | RFR | 5 | 4 | 5 | 5 | 5 | 5 | 1 | 1 | 5 | 5 |
|  | BayesA | 6 | 6 | 4 | 4 | 4 | 6 | 8 | 6 | 6 | 6 |
|  | BRR | 7 | 5 | 6 | 6 | 6 | 7 | 5 | 6 | 6 | 7 |
|  | BL | 8 | 7 | 7 | 7 | 7 | 8 | 7 | 8 | 8 | 8 |

PH, plant height; PCC, Pearson’s correlation coefficient; MAE, mean absolute error; MSE, mean squared error; RMSE, root mean squared error; R-squared, coefficient of determination R²; SRCC, Spearman's rank correlation coefficient; NDCG@5, top 5 normalized discounted cumulative gain; THR@10%, top 10% hit rate; BHR@70%, bottom 70% hit rate; CICE, combined index for correlation and error. rrBLUP, ridge regression best linear unbiased prediction; BL, Bayesian LASSO; BRR, Bayesian ridge regression; LightGBM, light gradient boosting machine; SVR, support vector regression; RF, random forest; DNNGP, deep neural network for genomic prediction.

**Supplementary Table S36.** Score and ranking of eight prediction methods on PCC and nine other evaluation metrics for the trait GW100 on the test set (20%) of the USNAM dataset.

|  | **Method** | **PCC** | **MAE** | **MSE** | **RMSE** | **R-squared** | **SRCC** | **NDCG@5** | **THR@10%** | **BHR@70%** | **CICE** |
| --- | --- | --- | --- | --- | --- | --- | --- | --- | --- | --- | --- |
| Score | rrBLUP | 0.709 | 1.615 | 4.205 | 2.051 | 0.499 | 0.703 | 0.931 | 0.511 | 0.853 | 0.816 |
|  | LightGBM | 0.673 | 1.683 | 4.623 | 2.150 | 0.449 | 0.666 | 0.930 | 0.500 | 0.843 | 0.796 |
|  | DNNGP | 0.660 | 3.323 | 17.798 | 4.219 | -1.120 | 0.658 | 0.921 | 0.400 | 0.818 | 0.754 |
|  | SVR | 0.622 | 1.922 | 5.878 | 2.424 | 0.300 | 0.608 | 0.840 | 0.433 | 0.826 | 0.765 |
|  | RFR | 0.482 | 2.187 | 7.407 | 2.722 | 0.118 | 0.485 | 0.786 | 0.378 | 0.797 | 0.689 |
|  | BayesA | 0.001 | 2.518 | 9.848 | 3.138 | -0.173 | 0.010 | 0.677 | 0.067 | 0.686 | 0.441 |
|  | BL | -0.003 | 2.490 | 9.634 | 3.104 | -0.148 | 0.009 | 0.649 | 0.067 | 0.674 | 0.440 |
|  | BRR | -0.003 | 2.472 | 9.504 | 3.083 | -0.132 | 0.018 | 0.652 | 0.056 | 0.682 | 0.441 |
| Ranking | rrBLUP | 1 | 1 | 1 | 1 | 1 | 1 | 1 | 1 | 1 | 1 |
|  | LightGBM | 2 | 2 | 2 | 2 | 2 | 2 | 2 | 2 | 2 | 2 |
|  | DNNGP | 3 | 8 | 8 | 8 | 8 | 3 | 3 | 4 | 4 | 4 |
|  | SVR | 4 | 3 | 3 | 3 | 3 | 4 | 4 | 3 | 3 | 3 |
|  | RFR | 5 | 4 | 4 | 4 | 4 | 5 | 5 | 5 | 5 | 5 |
|  | BayesA | 6 | 7 | 7 | 7 | 7 | 7 | 6 | 6 | 6 | 6 |
|  | BL | 7 | 6 | 6 | 6 | 6 | 8 | 8 | 6 | 8 | 8 |
|  | BRR | 8 | 5 | 5 | 5 | 5 | 6 | 7 | 8 | 7 | 7 |

GW100, 100 grain weight; PCC, Pearson’s correlation coefficient; MAE, mean absolute error; MSE, mean squared error; RMSE, root mean squared error; R-squared, coefficient of determination R²; SRCC, Spearman's rank correlation coefficient; NDCG@5, top 5 normalized discounted cumulative gain; THR@10%, top 10% hit rate; BHR@70%, bottom 70% hit rate; CICE, combined index for correlation and error. rrBLUP, ridge regression best linear unbiased prediction; BL, Bayesian LASSO; BRR, Bayesian ridge regression; LightGBM, light gradient boosting machine; SVR, support vector regression; RF, random forest; DNNGP, deep neural network for genomic prediction.

**Supplementary Table S37.** Score and ranking of eight prediction methods on PCC and nine other evaluation metrics for the trait PH on the test set (20%) of the USNAM dataset.

|  | **Method** | **PCC** | **MAE** | **MSE** | **RMSE** | **R-squared** | **SRCC** | **NDCG@5** | **THR@10%** | **BHR@70%** | **CICE** |
| --- | --- | --- | --- | --- | --- | --- | --- | --- | --- | --- | --- |
| Score | rrBLUP | 0.788 | 7.693 | 94.785 | 9.736 | 0.620 | 0.784 | 0.931 | 0.544 | 0.859 | 0.864 |
|  | DNNGP | 0.775 | 25.656 | 1052.479 | 32.442 | -3.218 | 0.768 | 0.932 | 0.544 | 0.849 | 0.796 |
|  | LightGBM | 0.752 | 8.288 | 109.028 | 10.442 | 0.563 | 0.748 | 0.909 | 0.511 | 0.851 | 0.844 |
|  | SVR | 0.736 | 8.805 | 124.078 | 11.139 | 0.503 | 0.727 | 0.927 | 0.467 | 0.842 | 0.834 |
|  | RFR | 0.411 | 11.876 | 217.796 | 14.758 | 0.127 | 0.457 | 0.882 | 0.256 | 0.772 | 0.661 |
|  | BRR | 0.109 | 13.062 | 260.893 | 16.152 | -0.046 | 0.080 | 0.821 | 0.011 | 0.696 | 0.506 |
|  | BL | 0.085 | 13.237 | 268.270 | 16.379 | -0.075 | 0.040 | 0.844 | 0.044 | 0.686 | 0.493 |
|  | BayesA | 0.069 | 13.393 | 275.204 | 16.589 | -0.103 | 0.024 | 0.825 | 0.044 | 0.683 | 0.485 |
| Ranking | rrBLUP | 1 | 1 | 1 | 1 | 1 | 1 | 2 | 1 | 1 | 1 |
|  | DNNGP | 2 | 8 | 8 | 8 | 8 | 2 | 1 | 1 | 3 | 4 |
|  | LightGBM | 3 | 2 | 2 | 2 | 2 | 3 | 4 | 3 | 2 | 2 |
|  | SVR | 4 | 3 | 3 | 3 | 3 | 4 | 3 | 4 | 4 | 3 |
|  | RFR | 5 | 4 | 4 | 4 | 4 | 5 | 5 | 5 | 5 | 5 |
|  | BRR | 6 | 5 | 5 | 5 | 5 | 6 | 8 | 8 | 6 | 6 |
|  | BL | 7 | 6 | 6 | 6 | 6 | 7 | 6 | 6 | 7 | 7 |
|  | BayesA | 8 | 7 | 7 | 7 | 7 | 8 | 7 | 6 | 8 | 8 |

PH, plant height; PCC, Pearson’s correlation coefficient; MAE, mean absolute error; MSE, mean squared error; RMSE, root mean squared error; R-squared, coefficient of determination R²; SRCC, Spearman's rank correlation coefficient; NDCG@5, top 5 normalized discounted cumulative gain; THR@10%, top 10% hit rate; BHR@70%, bottom 70% hit rate; CICE, combined index for correlation and error. rrBLUP, ridge regression best linear unbiased prediction; BL, Bayesian LASSO; BRR, Bayesian ridge regression; LightGBM, light gradient boosting machine; SVR, support vector regression; RF, random forest; DNNGP, deep neural network for genomic prediction.

**Supplementary Table S38.** Score and ranking of eight prediction methods on PCC and nine other evaluation metrics for the trait MSH on the test set (20%) of the millet827 dataset.

|  | **Method** | **PCC** | **MAE** | **MSE** | **RMSE** | **R-squared** | **SRCC** | **NDCG@5** | **THR@10%** | **BHR@70%** | **CICE** |
| --- | --- | --- | --- | --- | --- | --- | --- | --- | --- | --- | --- |
| Score | LightGBM | 0.366 | 10.818 | 177.404 | 13.319 | 0.110 | 0.338 | 0.822 | 0.250 | 0.785 | 0.632 |
|  | DNNGP | 0.298 | 18.167 | 511.830 | 22.624 | -1.567 | 0.262 | 0.820 | 0.250 | 0.776 | 0.567 |
|  | SVR | 0.252 | 11.971 | 224.349 | 14.978 | -0.125 | 0.212 | 0.815 | 0.250 | 0.759 | 0.570 |
|  | rrBLUP | 0.251 | 11.128 | 191.121 | 13.825 | 0.042 | 0.206 | 0.853 | 0.250 | 0.759 | 0.574 |
|  | RFR | 0.215 | 11.043 | 193.466 | 13.909 | 0.030 | 0.118 | 0.876 | 0.313 | 0.750 | 0.556 |
|  | BayesA | 0.069 | 12.149 | 238.888 | 15.456 | -0.198 | 0.087 | 0.805 | 0.125 | 0.698 | 0.478 |
|  | BL | 0.064 | 12.142 | 238.219 | 15.434 | -0.195 | 0.083 | 0.790 | 0.125 | 0.698 | 0.476 |
|  | BRR | 0.020 | 11.556 | 220.101 | 14.836 | -0.104 | 0.028 | 0.736 | 0.188 | 0.690 | 0.456 |
| Ranking | LightGBM | 1 | 1 | 1 | 1 | 1 | 1 | 3 | 2 | 1 | 1 |
|  | DNNGP | 2 | 8 | 8 | 8 | 8 | 2 | 4 | 2 | 2 | 4 |
|  | SVR | 3 | 5 | 5 | 5 | 5 | 3 | 5 | 2 | 3 | 3 |
|  | rrBLUP | 4 | 3 | 2 | 2 | 2 | 4 | 2 | 2 | 3 | 2 |
|  | RFR | 5 | 2 | 3 | 3 | 3 | 5 | 1 | 1 | 5 | 5 |
|  | BayesA | 6 | 7 | 7 | 7 | 7 | 6 | 6 | 7 | 6 | 6 |
|  | BL | 7 | 6 | 6 | 6 | 6 | 7 | 7 | 7 | 6 | 7 |
|  | BRR | 8 | 4 | 4 | 4 | 4 | 8 | 8 | 6 | 8 | 8 |

MSH, main stem height; PCC, Pearson’s correlation coefficient; MAE, mean absolute error; MSE, mean squared error; RMSE, root mean squared error; R-squared, coefficient of determination R²; SRCC, Spearman's rank correlation coefficient; NDCG@5, top 5 normalized discounted cumulative gain; THR@10%, top 10% hit rate; BHR@70%, bottom 70% hit rate; CICE, combined index for correlation and error. rrBLUP, ridge regression best linear unbiased prediction; BL, Bayesian LASSO; BRR, Bayesian ridge regression; LightGBM, light gradient boosting machine; SVR, support vector regression; RF, random forest; DNNGP, deep neural network for genomic prediction.

**Supplementary Table S39.** Score and ranking of eight prediction methods on PCC and nine other evaluation metrics for the trait PGW on the test set (20%) of the millet827 dataset.

|  | **Method** | **PCC** | **MAE** | **MSE** | **RMSE** | **R-squared** | **SRCC** | **NDCG@5** | **THR@10%** | **BHR@70%** | **CICE** |
| --- | --- | --- | --- | --- | --- | --- | --- | --- | --- | --- | --- |
| Score | DNNGP | 0.303 | 18.204 | 378.262 | 19.449 | -12.979 | 0.263 | 0.688 | 0.250 | 0.750 | 0.448 |
|  | rrBLUP | 0.261 | 3.985 | 25.515 | 5.051 | 0.057 | 0.208 | 0.624 | 0.250 | 0.741 | 0.576 |
|  | LightGBM | 0.222 | 4.200 | 27.355 | 5.230 | -0.011 | 0.211 | 0.520 | 0.188 | 0.759 | 0.554 |
|  | SVR | 0.190 | 4.620 | 33.685 | 5.804 | -0.245 | 0.170 | 0.541 | 0.188 | 0.733 | 0.533 |
|  | RFR | 0.188 | 4.022 | 26.527 | 5.150 | 0.020 | 0.145 | 0.651 | 0.188 | 0.724 | 0.539 |
|  | BL | -0.140 | 4.724 | 37.941 | 6.160 | -0.402 | -0.121 | 0.352 | 0.000 | 0.681 | 0.366 |
|  | BayesA | -0.144 | 4.862 | 40.000 | 6.325 | -0.478 | -0.115 | 0.311 | 0.000 | 0.690 | 0.363 |
|  | BRR | -0.156 | 4.486 | 34.105 | 5.840 | -0.260 | -0.144 | 0.352 | 0.000 | 0.681 | 0.362 |
| Ranking | DNNGP | 1 | 8 | 8 | 8 | 8 | 1 | 1 | 1 | 2 | 5 |
|  | rrBLUP | 2 | 1 | 1 | 1 | 1 | 3 | 3 | 1 | 3 | 1 |
|  | LightGBM | 3 | 3 | 3 | 3 | 3 | 2 | 5 | 3 | 1 | 2 |
|  | SVR | 4 | 5 | 4 | 4 | 4 | 4 | 4 | 3 | 4 | 4 |
|  | RFR | 5 | 2 | 2 | 2 | 2 | 5 | 2 | 3 | 5 | 3 |
|  | BL | 6 | 6 | 6 | 6 | 6 | 7 | 6 | 6 | 7 | 6 |
|  | BayesA | 7 | 7 | 7 | 7 | 7 | 6 | 8 | 6 | 6 | 7 |
|  | BRR | 8 | 4 | 5 | 5 | 5 | 8 | 6 | 6 | 7 | 8 |

PGW, per plant grain weight; PCC, Pearson’s correlation coefficient; MAE, mean absolute error; MSE, mean squared error; RMSE, root mean squared error; R-squared, coefficient of determination R²; SRCC, Spearman's rank correlation coefficient; NDCG@5, top 5 normalized discounted cumulative gain; THR@10%, top 10% hit rate; BHR@70%, bottom 70% hit rate; CICE, combined index for correlation and error. rrBLUP, ridge regression best linear unbiased prediction; BL, Bayesian LASSO; BRR, Bayesian ridge regression; LightGBM, light gradient boosting machine; SVR, support vector regression; RF, random forest; DNNGP, deep neural network for genomic prediction.

**Supplementary Table S40.** Comparison of scores and rankings of four prediction models on PCC and other evaluation metrics based on the example data in Supplementary Table S39.

|  | **Method** | **PCC** | **MAE** | **RMSE** | **R-squared** | **CICE** |
| --- | --- | --- | --- | --- | --- | --- |
| Score | Model 1 | 0.8345 | 1.28 | 2.11 | 0.46 | 0.851 |
|  | Model 2 | 0.8785 | 11.4 | 11.49 | -15.00 | 0.580 |
|  | Model 3 | 0.8978 | 20.2 | 20.20 | -48.48 | 0.502 |
|  | Model 4 | 0.9229 | 26.6 | 26.70 | -85.42 | 0.487 |
| Ranking | Model 1 | 4 | 1 | 1 | 1 | 1 |
|  | Model 2 | 3 | 2 | 2 | 2 | 2 |
|  | Model 3 | 2 | 3 | 3 | 3 | 3 |
|  | Model 4 | 1 | 4 | 4 | 4 | 4 |

PCC, Pearson’s correlation coefficient; MAE, mean absolute error; MSE, mean squared error; RMSE, root mean squared error; R-squared, coefficient of determination R²; CICE, combined index for correlation and error.

**Supplementary Table S41.** Advantages, disadvantages, and typical application scenarios of some commonly used evaluation metrics.

| **Metric** | **Advantages** | **Disadvantages** | **Applicability in different scenarios** |
| --- | --- | --- | --- |
| PCC | - Linear relationship quantification: PCC effectively measures the strength and direction of a linear association between predicted and observed values, providing a standardized value that indicates perfect negative correlation, no correlation, or perfect positive correlation. - Ease of interpretation: The coefficient's value directly conveys the degree of correlation, making it straightforward to communicate and compare across different models. - Insensitive to scale changes: PCC is not easily affected by linear transformations (e.g., scaling, shifting) of the variables being correlated. | - Poor measurement of non-linear relationships: PCC may provide misleading results if the relationship between variables is non-linear. - Sensitivity to test set size and outliers: Different sizes of test sets or outliers may significantly affect the evaluation scores, potentially distorting the overall assessment. - Hypothesis of normality and homoscedasticity (Rainio et al, 2024): PCC's validity relies on the assumption that both variables follow a bivariate normal distribution and have constant variance across their range. | Applicable to correlation analysis such as genotype-phenotype association studies, gene–environment interaction analysis. |
| MAE | - Ease of interpretation (Mitchell P.L., 1997): Its simplicity and straightforward calculation make it easy for plant breeders and researchers, who may not be statisticians, to understand and communicate model performance. - Robustness to outliers and test set size: MAE has low sensitivity to outliers and test set size, ensuring a more stable evaluation of the model's predictive ability. - Unit consistency: MAE retains the same units as the measured traits (e.g., yield in kg/ha), enabling direct comparison with industry standards, breeding targets, or economic thresholds. | - Ignorance of error direction: In some scenarios, overestimating or underestimating a trait value may have distinct consequences. For example, overestimating disease resistance could lead to the release of susceptible cultivars, while underestimating yield potential might result in missed opportunities for crop improvement. - ﻿Scale dependency: Its sensitivity to scale can make comparisons across traits or datasets challenging, necessitating normalization or standardization procedures to ensure fair assessments. | Applicable to accurate prediction of trait values such as crop yield estimation and prediction, trait prediction from high-throughput phenotyping. |
| MSE | - Sensitivity to large errors: MSE penalizes large errors more severely than small ones due to its squaring of the differences. This sensitivity makes MSE a useful metric when accurate predictions are crucial, especially for the traits that have significant impact on plant performance.﻿ - Easy to interpret and compare: MSE provides a numerical value that can be easily compared across different models or datasets. This allows breeders to quickly identify the best-performing models based on their MSE values. | - Unit inconsistency: MSE is expressed in squared units of the target trait, which can be less intuitive and harder to compare with practical breeding targets or economic thresholds. To restore unit consistency, researchers may need to take some transformations. - Sensitivity to outliers: MSE is more sensitive to outliers than MAE, as squaring the errors amplifies their impact on the overall error measure. This increased sensitivity may lead to unstable or misleading model evaluations.﻿﻿ | Similar to MAE. |
| RMSE | - Sensitivity to large errors: This feature is similar to MSE. - Improved interpretability: RMSE provides a more intuitive measure of prediction accuracy than MSE, as it represents the average error magnitude on the same scale as the measured trait. - Comparability across traits: Since RMSE is scaled to the original units of the target variable, it allows for easier comparison of prediction errors across different traits with varying scales. | Its disadvantages are the same as MSE. | Similar to MAE and MSE. |
| R² score | - Intuitive interpretation: The R² score provides a straightforward measure of how well a model fits the data. This makes it easy for breeders to quickly assess the predictive power of a model. - Comparison across models: R² allows breeders to compare the performance of different models on the same dataset. It serves as a standard metric for evaluating model accuracy, making it easy to identify the best-fitting model for a particular breeding problem. | - Sensitivity to scaling: R² is sensitive to the scale of the data. If the target variable is scaled differently, the R² score may change even if the relationship between predictor and target variables remains the same. - Sensitive to outliers: Like metrics such as MSE and RMSE, R² score is equally sensitive to outliers. - Unable to determine the causal relationship of the model: Although R² can explain the proportion of variation in the dependent variable, it cannot determine the causal relationship between the dependent variable and the independent variable. | More suitable for linear regression models, but less appropriate for models with nonlinear and complex relationships |
| SRCC | - Non-parametric nature: Unlike PCC, which assumes a linear relationship and normal distribution of the variables, SRCC does not require these assumptions. It is robust against outliers, skewed distributions, and non-linear relationships, making it suitable for various types of plant breeding data that might deviate from these assumptions. - ﻿Ordinal data compatibility: Plant breeding often involves the assessment of traits with ordinal scales (e.g., disease resistance ratings from 1 to 5) or subjective evaluations. SRCC can effectively handle such data, as it only considers the rank of observations rather than their values. - ﻿﻿Ease of interpretation: Similar to PCC, allowing for straightforward interpretation and comparison across studies. | - Loss of information: Converting raw data to ranks discards information about the magnitude of differences between variables. This may be problematic when subtle variations in trait values are crucial for plant breeding decisions. - Sensitivity to ties: In cases where multiple observations share the same rank, Spearman's correlation can become less reliable. Ties are more likely to occur in small sample sizes or when measuring traits with limited variability, potentially leading to biased estimates of the correlation. - Assumption of monotonicity: SRCC assumes a monotonic relationship between the two variables, meaning their ranks either consistently increase or decrease together. If the relationship is non-monotonic (e.g., U-shaped or inverted U-shaped), the coefficient may not accurately reflect the true association. | It is suitable for models that evaluate the degree of correlation between observed and predicted values in terms of ranking rather than their values, e.g. categorical or ordinal predictions. |
| NDCG@K | - Ranking sensitivity: NDCG@K effectively captures the quality of the top-ranked results. It assigns higher weights to items at the top of the list, reflecting the user's tendency to focus on the first few items. - Normalization: By normalizing the discounted cumulative gain values, it allows for comparison across different queries or user sessions, even when the total number of relevant items differs. | - Sensitivity to K: The choice of K can significantly influence the score. Different values of K may lead to different rankings, making it challenging to compare systems using different K values. - Focus on top items: While its emphasis on top items is advantageous in many scenarios, it may provide limited insight into the quality of items ranked lower in the list. This can be problematic in scenarios where users explore beyond the top few items. | It is suitable for prediction models that that focus on the top K items ranking. |
| THR@P% | - Ease of interpretation: It provides a simple and intuitive measure of a model's ability to identify relevant items within the top-ranked percentage. Thus, it is easy to understand and communicate.﻿ - Focus on high-ranking items: By focusing on the top-ranked items, it emphasizes the importance of accurate ranking, particularly for scenarios where users are likely to only consider a small subset of the recommended items.﻿ - Flexible threshold: It allows for flexibility in defining the threshold. This makes it suitable for scenarios where the number of relevant items may vary or where different thresholds are of interest. | - Sensitive to threshold selection: The performance of the top P% hit rate is highly dependent on the choice of the threshold P. Selecting an inappropriate threshold may fail to capture the model's true performance. - Lack of granularity: It provides a single aggregate value for the entire top P% of the ranked list, without distinguishing between the relative positions of relevant items within this portion. - Limited information: The top P% hit rate only considers the items within the top P% and ignores the rest. This can be limiting in scenarios where the ranking of items beyond the top P% is also important. | It is more suitable for prediction tasks that focus on the top P% rankings. |
| BHR@P% | Similar to the metric THR@P%, but with the difference that BHR@P% focuses on the hit rate at the tail. | Similar to the metric THR@P%, but with the difference that BHR@P% focuses on the hit rate at the tail. | It is suitable for prediction tasks that focus on the bottom P% rankings. |

PCC, Pearson’s correlation coefficient; MAE, mean absolute error; MSE, mean squared error; RMSE, root mean squared error; R-squared, coefficient of determination R²; SRCC, Spearman's rank correlation coefficient; NDCG@K, top K normalized discounted cumulative gain; THR@P%, top P% hit rate; BHR@P%, bottom P% hit rate.

**Supplementary Table S42.** Guidelines and detailed steps on how to select and apply evaluation metrics in some typical scenarios.

| **Application scenario** | **Scenario description** | **Candidate evaluation scheme and its detailed instructions** | **Suggested evaluation metrics** |
| --- | --- | --- | --- |
| Crop yield prediction | Using multi-omics data such as genotype and environmental data to predict the yield potential of crops under different environments. | Use a combination of PCC with any one from MAE, RMSE, R², or RMSD.  One can use any of MAE, RMSE, and RMSD to evaluate prediction errors, use R^2^ to evaluate the predictive ability of the model, and employ the PCC to measure the linear correlation between predicted and observed values. | CICE |
| Selection of elite lines | Select the top-K individuals or eliminate the bottom-K individuals in the ranking. | Do any of the following: (1) THR@P%; (2) NDCG@K; (3) SRCC; (4) A combination of PCC with any one from MAE, RMSE, R², or RMSD.  If the user focuses on the top-K individuals, it is recommended to use schemes (1) and (2); Otherwise, use schemes (3) and (4). | THR@P% |
| Estimates of genetic and phenotypic correlation | Identify the statistically significant correlation between genomes and phenotypic traits. | Use PCC, or SPCC.  When the data does not follow a normal distribution, or the variables are ordered and there is a monotonic relationship between the variables, it is suggested to use the SPCC; Otherwise, use PCC. | If the data follows a normal distribution, PCC is used; Otherwise, SPCC is used. |
| Screening of potential promotion areas for new varieties | The phenotype prediction values of new varieties in each region receive more attention, and the prediction errors of the model is as small as possible. | Use a combination of PCC and MAE, or use NDCG@K as a supplement. Use MAE to evaluate prediction errors, and use the PCC to measure the linear correlation between predicted values and actual observed values. If only the top K varieties or regions are selected, NDCG@K can be used as a supplement. | CICE |
| Analysis of genotype×environment interaction | The metrics should comprehensively reflect the performance of the model in dealing with complex traits and environmental impacts | Use a combination of PCC and MAE.  Use PCC measures the linear correlation, MAE provides an indication of the prediction error of the model. | CICE |
| **Detailed steps on how to select and apply evaluation metrics** | | | |
| **Principles for selecting evaluation metrics:**  For application scenarios with correlation analysis as the main objective, priority should be given to using indicators such as PCC or SPCC; For application scenarios with the main goal of minimizing prediction errors, priority is given to the scores of indicators such as MAE, RMSE, RMSD, etc., with PCC scores as an auxiliary factor; For application scenarios aimed at screening top individuals, indicators such as NDCG and THR can be used for measurement.  **Detailed steps for model evaluation:**  (1) Clarify the application scenario and prediction objectives of the model, and analyze the data characteristics such as data distribution, dataset size, and data source of the quantitative traits to be predicted.  (2) Preprocess the data by handling missing values, outliers, and transformation or standardization, and select the appropriate model for the task.  (3) Referring to the evaluation metrics used in typical application scenarios, choose suitable assessment metrics, and then evaluate the model using k-fold cross-validation.  (4) Continuously iterate and optimize the model based on scores of all evaluation metrics and the visual evaluation by drawing the scatter plot of predicted values and observed values.  **Notes:**  (1) To estimate the generalization performance of different models, a k-fold cross-validation scheme is recommended to conduct.  (2) To ensure fair comparison, it should be ensured that all methods use the same split. Evaluation scores were computed for k cross-validation iterations and averaged.  (3) If the PCC evaluation metric is used, a Student’s T-Test can be employed to assess the significance of the PCC, and the formula is: $t=r\sqrt{\frac{n-2}{1-r^{2}}}$. Among them, *n* is the number of samples, *r* is the value of PCC, and *t* is the test value.  (4) Visual evaluation such as drawing a scatter plot of predicted values versus observed values is a valuable supplement. | | | |

PCC, Pearson’s correlation coefficient; MAE, mean absolute error; MSE, mean squared error; RMSE, root mean squared error; R² score, coefficient of determination of the model; SRCC, Spearman's rank correlation coefficient; NDCG@K, top-K normalized discounted cumulative gain (Blondel et al., 2015); THR@P%, top-P percent hit ratio; BHR@P%, bottom-P percent hit ratio; RMSD, root mean squared deviation (Piñeiro et al., 2008) ; CICE, combined index for correlation and error.

1. **Supplementary References**
2. Blondel, M., Onogi, A., Iwata, H., and Ueda, N. (2015). A Ranking Approach to Genomic Selection. Plos One 10: e0128570
3. Buckler, E.S., Holland, J.B., Bradbury, P.J., Acharya, C.B., Brown, P.J., Browne, C., Ersoz, E., Flint-Garcia, S., Garcia, A., Glaubitz, J.C., et al. (2009). The Genetic Architecture of Maize Flowering Time. Science 325:714-718. DOI: 10.1126/science.1174276.
4. Garcia, M., Eckermann, P., Haefele, S., Satija, S., Sznajder, B., Timmins, A., Baumann, U., Wolters, P., Mather, D.E., and Fleury, D. (2019). Genome-wide association mapping of grain yield in a diverse collection of spring wheat (Triticum aestivum L.) evaluated in southern Australia. PLoS One 14: e0211730. DOI: 10.1371/journal.pone.0211730
5. Li J, Zhang D, Yang F, Zhang Q, Pan S, Zhao X, Zhang Q, Han Y, Yang J, Wang K, Zhao C. (2024) TrG2P: A transfer learning-based tool integrating multi-trait data for accurate prediction of crop yield. Plant Commun. 100975. DOI: 10.1016/j.xplc.2024.100975. (In Press)
6. McFarland, B.A., AlKhalifah, N., Bohn, M., Bubert, J., Buckler, E.S., Ciampitti, I., Edwards, J., Ertl, D., Gage, J.L., Falcon, C.M., et al. (2020). Maize genomes to fields (G2F): 2014-2017 field seasons: genotype, phenotype, climatic, soil, and inbred ear image datasets. BMC Res Notes 13:71. DOI:10.1186/s13104-020-4922-8.
7. McLaren, C.G., Bruskiewich, R.M., Portugal, A.M., and Cosico, A.B. (2005). The International Rice Information System. A platform for meta-analysis of rice crop data. Plant Physiol. 139:637–642.
8. Mitchell P.L. (1997). Misuse of regression for empirical validation of models. 54: 313-326. https://doi.org/10.1016/S0308-521X(96)00077-7.
9. Piñeiro, G., Perelman, S., Guerschman, J.P., and Paruelo, J.M. (2008). How to evaluate models: Observed vs. predicted or predicted vs. observed? Ecol. Model 216:316-322.
10. Rainio, O., Teuho, J., and Klén, R. (2024). Evaluation metrics and statistical tests for machine learning. Sci Rep 14, 6086. https://doi.org/10.1038/s41598-024-56706-x
11. Scikit-learn. (2023). Metrics and scoring: quantifying the quality of predictions. <https://scikit-learn.org/stable/modules/model_evaluation.html>. [Accessed 15 Dec 2023]. accessed on.
12. Spindel, J.E., Begum, H., Akdemir, D., Virk, P., Collard, B., Redoña, E., Atlin, G., Jannink, J., McCouch, S. (2015). Genomic Selection and Association Mapping in rice (Oryza sativa): Effect of trait genetic architecture, training population composition, marker number and statistical model on accuracy of rice genomic selection in elite, tropical rice breeding lines. PLoS Genetics 11(2). DOI: 10.1371/journal.pgen.1004982
13. Wang, K.L., Abid, M.A., Rasheed, A., Crossa, J., Hearne, S., and Li, H.H. (2023). DNNGP, a deep neural network-based method for genomic prediction using multi-omics data in plants. Mol. Plant 16:279-293.
14. Wang, Y., Wang, X., Sun, S. et al. GWAS, MWAS and mGWAS provide insights into precision agriculture based on genotype-dependent microbial effects in foxtail millet. Nat. Commun. 13, 5913 (2022). https://doi.org/10.1038/s41467-022-33238-4
15. Yang N, Lu Y, Yang X, Huang J, Zhou Y, Ali F, et al. (2014) Genome Wide Association Studies Using a New Nonparametric Model Reveal the Genetic Architecture of 17 Agronomic Traits in an Enlarged Maize Association Panel. PLoS Genet 10(9): e1004573. https://doi.org/10.1371/journal.pgen.1004573
